# Associations of arsenic exposure and folate in maternal leukocyte DNA methylation: a case-control study of mothers with spina-bifida affected children

**DOI:** 10.1186/s12940-025-01254-8

**Published:** 2026-01-16

**Authors:** Amy M. Inkster, Anne K. Bozack, Bernardo Lemos, Tabitha Lumour-Mensah, Sudipta Kumar Mukherjee, Shekh Muhammad Ekramullah, DM Arman, Joynul Islam, Xingyan Wang, Liming Liang, Richard H. Finnell, Maitreyi Mazumdar, Andres Cardenas

**Affiliations:** 1 Department of Epidemiology and Population Health, Stanford School of Medicine, Stanford, CA USA; 2 Department of Pharmacology and Toxicology, R. Ken Coit College of Pharmacy, University of Arizona, Tucson, AZ USA; 3 Coit Center for Longevity and Neurotherapeutics, University of Arizona, Tucson, AZ USA; 4 Department of Environmental Health, Harvard T.H. Chan School of Public Health, Boston, MA USA; 5 Department of Pediatric Neurosurgery, National Institute of Neurosciences & Hospital (NINS&H), Dhaka, Bangladesh; 6 Department of Clinical Neurosurgery, National Institute of Neurosciences and Hospital (NINS&H), Dhaka, Bangladesh; 7 Department of Epidemiology, Harvard T.H. Chan School of Public Health, Boston, MA USA; 8 Department of Molecular and Cellular Biology, Center for Precision Environmental Health, Baylor College of Medicine, Houston, TX USA; 9 Department of Molecular and Human Genetics, Department of Medicine, Baylor College of Medicine, Houston, TX USA; 10 Department of Neurology, Boston Children’s Hospital, Boston, MA USA; 11 Department of Neurology, Harvard Medical School, Boston, MA USA

**Keywords:** DNA methylation, Epigenetics, Arsenic, Folate, Spina bifida, Maternal, Bangladesh

## Abstract

**Background:**

In Bangladesh, more than a quarter of drinking water tubewells are contaminated with arsenic above the national standard (50 µg/l), while nearly half exceed the World Health Organization guideline (10 µg/l). Among other negative health consequences, arsenic is a suspected environmental risk factor for neural tube defects (NTDs), including spina bifida. Maternal folate status protects against NTDs, though recent evidence suggests arsenic attenuates folate’s protective effects. Arsenic is methylated prior to excretion with methyl groups produced in one-carbon metabolism, for which folate is a cofactor. We thus hypothesized that DNA methylation (DNAme) may provide insight into the interactions between arsenic, maternal folate levels, and offspring spina bifida.

**Methods:**

Here we analyzed leukocyte DNAme using the Illumina MethylationEPIC v2.0 array in 374 women from Bangladesh, 246 with a previous spina-bifida affected birth and 128 controls. Chronic arsenic exposure was evaluated in maternal toenail; fasting plasma folate was measured at blood draw. Linear models evaluated DNAme associated with offspring spina bifida, arsenic, folate, and their interaction terms.

**Results:**

Maternal DNAme was associated with spina bifida at 71 CpGs, arsenic at 6 CpGs, and folate at 33 CpGs (all FDR < 0.05). The spina bifida*arsenic and arsenic*folate interactions were associated with 11 and 28 CpGs, respectively, while spina bifida*folate returned no significant associations. We observed lower DNAme in mothers of spina bifida cases at significant loci, including in developmental genes such as *HOXB3* and *HOXB4*. Arsenic’s influence on DNAme was more pronounced in individuals with low folate.

**Conclusions:**

Our work suggests that postnatal maternal leukocyte DNAme is associated with offspring spina bifida status and is modified by arsenic exposure but not plasma folate.

**Supplementary Information:**

The online version contains supplementary material available at 10.1186/s12940-025-01254-8.

## Highlights

- Maternal DNA methylation is responsive to arsenic, folate, and offspring spina bifida.

- Offspring spina bifida affects > 50 postnatal maternal DNA methylation loci.

- DNA methylation at significant loci decreases with higher folate and with higher arsenic.

- Arsenic*spina bifida and arsenic*folate interactions affect maternal DNA methylation.

- Maternal DNA methylation is not significantly associated with folate*spina bifida.

**Supplementary Information:**

The online version contains supplementary material available at 10.1186/s12940-025-01254-8.

## Background

Neural tube defects (NTDs) including spina bifida and anencephaly are among the most common severe congenital anomalies, arising in the third to fourth week of gestation when the developing neural plate fails to elevate, fold, and close [1]. NTDs are complex disorders with both genetic and environmental risk factors [2, 3]. Large clinical trials in the late 20th century established that pre/perinatal maternal supplementation with folic acid, a stable, synthetic form of folate, exerted a clear protective effect against NTD development [4]. Several countries have implemented national folic acid fortification programs resulting in decreased NTD incidence among subsequent live births [5]. However, the global prevalence of NTDs remains high at approximately 2 per 10,000 live births, with rates as high as 10 per 10,000 disproportionately observed in low and middle-income countries [6–8]. The mechanism(s) by which folic acid fortification and maternal folate status reduce the risk of NTDs are not well understood, and the persistent burden of NTDs worldwide suggests the contribution of additional important factors in NTD pathogenesis.

Among suspected risk factors for NTDs are maternal environmental exposures such as pesticides [9–11], polycyclic aromatic hydrocarbons [12], and heavy metals [13–17]. Arsenic stands out as an environmental risk factor for NTDs, supported by evidence from animal models where arsenic treatment is associated with manifestation of neural tube defects and craniofacial malformations [18–20]. Population-based studies further indicate an association between environmental arsenic and rates of spina bifida [21–23]. Specifically in Bangladesh, exposure to arsenic-contaminated drinking water is chronic and widespread, with more than 70 million people exposed to arsenic in drinking water at exceedingly high concentrations [24, 25]. A nationwide water quality survey conducted between 1996 and 2010 indicated that 42% of tubewells tested in Bangladesh reported arsenic in excess of 10 µg/L, the World Health Organization (WHO) drinking water guideline value [26], while more than a quarter of all wells tested exceeded 50 µg/L [27]. Bangladesh also has a high prevalence of NTDs, estimated between 2.7 and 4.7 per 1,000 live births [6, 28, 29], with some series reporting rates as high as 8.9 per 1,000 births [30–32]. Of note, recent work in Bangladesh has indicated that arsenic may attenuate the protective effect of folic acid in NTD prevention, pointing towards a shared biological mechanism of action [33, 34].

The mechanistic link between maternal folate status, arsenic, and spina bifida may lie in one-carbon metabolism (OCM), the central metabolic pathway connecting folate status to downstream biology including arsenic and DNA methylation. Folate donates one-carbon units to OCM, fueling both the methylation cycle (producing the universal methyl donor, S-adenosyl methionine) and the DNA cycle (producing nucleotides) [35]. At the same time, OCM is the primary pathway for arsenic methylation, a metabolic process that decreases arsenic toxicity and increases its urinary excretion [36–38]. Genetic variants associated with reduced enzymatic activity of the OCM methylation cycle enzyme MTHFR have been variably observed at higher frequency in association with spina bifida [39] and may act via reducing one-carbon availability [40], implicating OCM as a key molecular link between these factors.

**Fig. 1:**
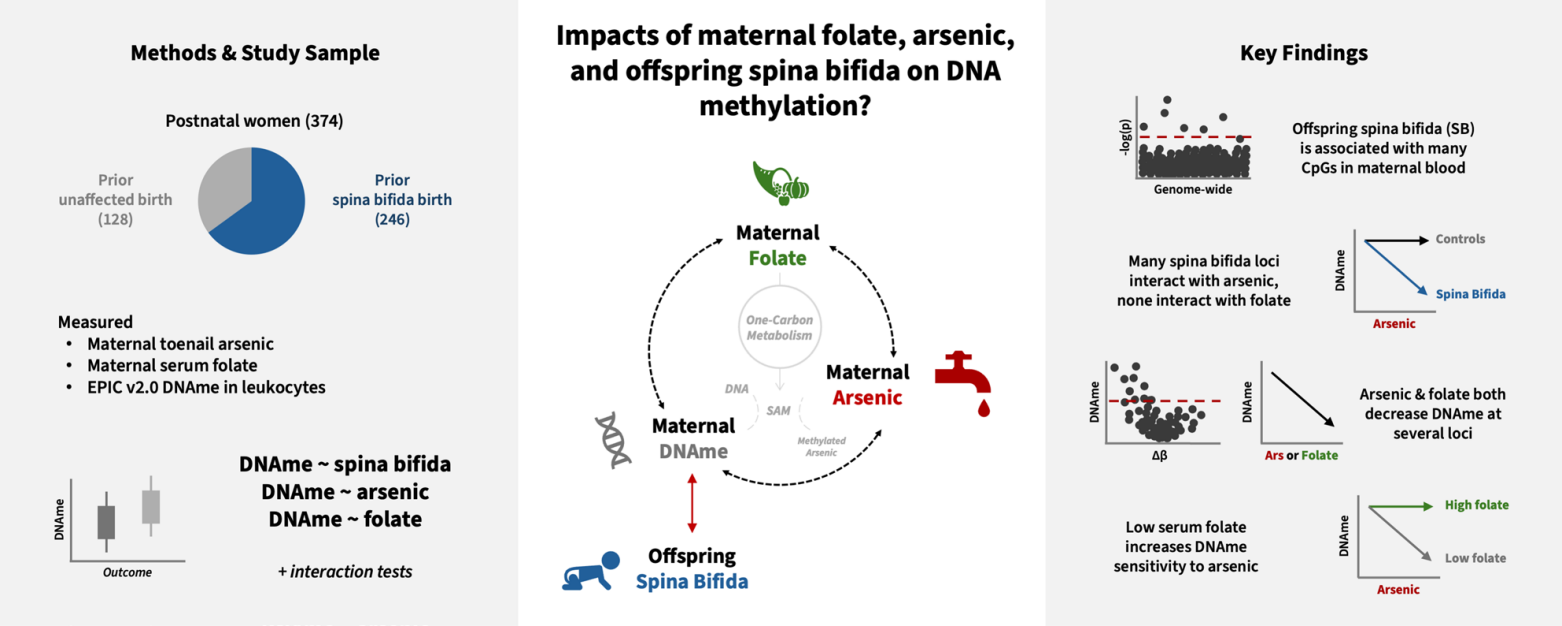
Graphical depiction of study design. DNAme refers to DNA methylation

DNA methylation (DNAme) is an essential epigenetic modification, and represents a potential molecular readout for understanding the connections between spina bifida pathogenesis, the protective role of folate, and the risk effects of arsenic. As a primary product of the methylation cycle of OCM, DNAme patterns may be influenced by both folate availability (as a methyl donor), and arsenic exposure (as a methyl consumer). Additionally, aberrant DNAme patterns have been identified in studies of spina bifida in humans, and in animal models of NTDs [41–45], making it a relevant molecular layer to investigate the etiology of spina bifida. However, how maternal arsenic exposure, folate status, and a spina bifida-affected pregnancy (i) individually influence maternal DNAme, and (ii) interface with each other at the level of DNAme remains poorly understood. Here, in an exploratory study conducted in a high-risk population from Bangladesh, we aimed to characterize the independent associations of arsenic exposure, folate status, and offspring spina bifida status with maternal leukocyte DNAme patterns. Additionally, we investigated two-way cross-product interactions between these factors to begin to characterize their complex molecular interplay, see Fig. 1. Ultimately, this work seeks to elucidate the biological pathways relating maternal health and exposures to spina bifida risk, providing a foundation for future mechanistic research.

## Methods

### Study population

This project includes participants from a larger study evaluating the relationship between spina bifida and environmental arsenic in Bangladesh [33, 46, 47]. This study and all protocols were reviewed and approved by the Bangladesh Medical Research Council (BMRC IRB registration number 006 23 08 2016; reference number BMRC/NREC/2016–2019/1651; approved 10 November 2016), the Human Research Committees at Boston Children’s Hospital (protocol number IRB-P00019768; approved 22 August 2015), the National Institutes of Neurosciences & Hospital (NINS&H) (ERC-NINS number 2016/07/10, approved August 1, 2016), and the Dhaka Shishu Hospital (No. Admin/1081/2018/DaShiHa, approved June 3, 2018); the Harvard Chan School ceded review to Boston Children’s Hospital. Clinical trial number: not applicable. Written informed consent was obtained from all participants, and all procedures complied with relevant legislation and institutional protocols. Mothers of children under one year of age with spina bifida (verified by a neurosurgeon and imaging) were recruited from the NINS&H between December 2016 and December 2022. Controls were mothers of similarly aged children recruited at NINS&H during the same time period, and from the adjacent Dhaka Shihsu Hospital starting in June 2018. Exclusion criteria included maternal anticonvulsant medication use, chromosomal abnormality (Trisomy 13 or 18) in the child, and cancer or keratosis in the child (i.e., conditions believed to be related to arsenic exposure). The larger study enrolled 514 individuals: 333 with infants affected by spina bifida and 165 mothers of unaffected controls. For the DNAme study, a subset of 381 individuals provided leukocyte samples at the study enrollment hospital visit, including 251 mothers of spina bifida cases and 130 mothers of controls. There were fewer control mothers enrolled as biological sample collection from controls was suspended during the COVID-19 pandemic.

### Arsenic and folate measurements

Arsenic was quantified in the maternal toenail as previously described [47]. In brief, nail samples from all ten toes were collected at the enrollment visit; arsenic concentration was assessed using inductively coupled plasma mass spectrometry (ICP-MS) at the Dartmouth College Trace Element Analysis Core Facility Laboratory. Arsenic measurements for 15 participants were not available and were imputed to the median of the overall study sample, 0.466 µg/g (all participants missing data were mothers of cases, missingness was random and due to amount of nail available),

For folate assessment, blood samples (minimum 8 h fasting for glucose assessment) were collected at the enrollment visit and were centrifuged without anticoagulant to isolate serum, as previously described [47]. Folate was measured in serum aliquots at the NINS&H using a Chemiluminescent Microparticle Immunoassay (CMIA) with an ARCHITECT c4000 (Abbott Company, Abbott Park, IL, USA).

### DNA methylation data processing

Plasma was isolated by centrifugation from whole blood samples at NINS&H, after which the buffy coat layer was aspirated and frozen at −20 °C and shipped to Baylor University (Waco TX) where they were stored at −80 °C. DNA was extracted and shipped to the University of Minnesota Genomics Center (UMGC) for array processing including PicoGreen quality assessment, bisulfite conversion using the Zymo DNA Methylation kit (Zymo Research, Irvine CA), and randomization of bisulfite-converted samples across six array batches according to key study variables including offspring spina bifida status. DNA methylation was assessed using the Illumina Infinium MethylationEPIC v2.0 bead chip.

Raw IDAT files were read into R and the *ewastools* and *minfi* packages were used to assess sample quality [48, 49]. Quality checks included assessment of fluorescence intensity, median inter-array correlation, control probes, and in-built genotyping “rs” probes. Sample genetic identity was confirmed to be unique using the 65 rs probes, except in the case of known technical replicates (*n* = 11), which were removed along with four leukocyte samples removed for being unexpected duplicates based on SNP genotypes. Sample sex was assessed from the data directly based on X and Y chromosome fluorescence intensity in the method of the *ewastools* package [49]; for all leukocyte samples, data-derived sex corresponded with reported female sex. Three additional leukocyte samples were removed for being flagged in two or more of the following quality checks: *minfi getQC* function failure, failure in any of the 17 control probes, total fluorescence intensity more than 3 standard deviations below the mean of the dataset, inter-array correlation < 0.90, SNP outlier log odds metric > −4, > 1% of probes failing either detection P value or bead count assessments, orr average detection P value > 0.05. Of these three excluded samples, two failed the SNP outlier logodds check and had > 1% failed probes; the third failed all of the following and was removed: *minfi getQC*, control probes, fluorescence intensity, inter-array correlation, SNP outlier logodds, > 1% failed probes, and average detection P value.

Data were background and dye-bias corrected using the *preprocessNoob* function and normalized with *preprocessQuantile* [48]. M-values were corrected using *sva ComBat* [50] for EPIC array batch (96-well array processing plate). Replicate CpG probes from the v2.0 array were collapsed to the single representative probe with the lowest mean detection P value across all samples. CpG probes were then filtered in the following order to exclude (i) CpGs with detection P value > 0.01 or beadcount < 3 in > 1% of samples, (ii) CpGs mapping to the X or Y chromosome, (iii) CpGs indexed as polymorphic, cross-hybridizing, or otherwise masked in the Kaur et al. (2024) or Peters et al. (2024) annotations [51, 52].

After data processing and quality control, 374 maternal leukocyte samples remained (246 mothers of spina bifida cases, 128 mothers of controls), with DNAme data at 837,569 unique CpGs. The proportions of six major blood cell types (B cells, CD4T, CD8T, Monocytes, Neutrophils, and NK cells) were estimated from normalized DNAme beta values using the IDOL reference and the *FlowSorted.Blood.EPIC* package [53, 54] with CpG probes missing on the v2.0 array removed from the data and reference set prior to estimation.

### Epigenome-wide association studies

Multi-step epigenome-wide association studies were conducted using adjusted linear regression models to identify differentially methylated CpGs associated with offspring spina bifida status, arsenic exposure, and folate levels. Arsenic and folate were analyzed on their original scales without transformation. We additionally tested for effect modification of spina bifida status by arsenic or by folate, as well as effect modification of arsenic by folate, using cross-product interaction terms. All models were run on DNAme M-values using the *limma* package in R [55], effect sizes in Δβ units were computed using the M-model-coef method [56]. All models were adjusted for maternal age (years), BMI, education (</≥ university), and DNAme-estimated cell-type proportions (B cells, CD4T, CD8T, monocytes, natural killer cells, and neutrophils) and EPIC array row (8 levels). Multiple comparisons were accounted for using both a false discovery rate (FDR) and Bonferroni approach.

A single marginal model was used to evaluate the main effects of spina bifida status (Model 1a), toenail arsenic (Model 1b), and plasma folate (Model 1c), from this model we extracted the results for each of the three main effects one at a time, representing their associations with DNAme while mutually adjusting for each other. Cross-product interaction terms were then added one at a time to the marginal model to investigate effect modification of spina bifida status by arsenic (Model 2, spina bifida*arsenic), spina bifida status by folate (Model 3, spina bifida*folate), and arsenic by folate (Model 4, arsenic*folate). For EWAS models with significant interaction term findings, we conducted secondary analyses stratified by spina bifida or folate status (categorized as < 4 ng/mL or 4 ng/mL based on folate sufficiency thresholds [57]); in these follow-up analyses, a nominal p-value < 0.05 was considered significant.

Differentially methylated regions (DMRs) were evaluated with the same structure of linear regression models described above. The *DMRcate* package version 3.2.1 was utilized for compatibility with the v2.0 array [58], replicate probe-collapsed M-values were provided as input with the lambda parameter set to 1000 base pairs, the scaling factor for bandwidth “C” value set to 2, and “differential” selected as the analysis type. DMRcate 3.2.1 uses the FDR method to account for multiple comparisons after combining raw p values from individual CpGs using the Stouffer method.

All statistical analyses were carried out using R version 4.4.2. Genomic coordinates of all results are communicated relative to genome build hg38 unless otherwise specified.

### Gene-set enrichment analysis

Gene-set enrichment analysis was performed on CpGs reaching FDR < 0.05 in individual epigenome-wide association studies against the KEGG and GO databases using the *gometh* function from the *missMethyl* package using default settings [59].

### Literature candidate lookup analysis

To determine whether differentially methylated CpGs identified in the current study replicated previously published results, we performed a lookup analysis, comparing CpGs at FDR and nominal (*p* < 0.05) significance in our study with CpG sites previously associated with neural tube defects, arsenic exposure, and folate.

## Results

### Participant characteristics

Participant characteristics for the 374 individuals included in the analysis are presented in Table 1. All participants were female and had either given birth to a spina bifida-affected child (*n* = 246) or a control child (*n* = 128) in the months prior to blood collection. Mothers of spina bifida-affected children had lower average levels of education (94.7% less than university) than mothers of controls (87.5% less than university) (*p* = 0.023), and the spina bifida-affected children were more often female (49.2% female spina bifida-affected offspring versus 35.2% female control offspring, *p* = 0.013). Maternal blood collection also occurred sooner postnatally in the mothers of spina bifida-affected children (75.44 ± 85.2 days) than in the mothers of controls (187.23 ± 97.4 days). The mean estimated proportion of natural killer cells was marginally lower in mothers of spina bifida-affected children (0.04845 ± 0.0187) than mothers of controls (0.05385 ± 0.0215) (*p* = 0.013); no other cell type proportion or demographic characteristics differed significantly by case-control status; cell type proportions were included as covariates in downstream statistical models. Participants were evenly distributed across the six EPIC v2.0 array processing batches.

**Table 1:** Participant characteristics among 374 Bangladeshi women with leukocyte DNAme profiled by the EPIC v2.0 array. The p value column reflects unadjusted comparison of women with spina bifida births to those with control births, non-significant results (*p* > 0.05) are indicated with a Dash (-). SD refers to standard deviation

Sociodemographic and technical characteristics	Spina bifida birth (case) *N* = 246	Non-spina bifida birth (control) *N* = 128	*p* value*
	Mean (SD) or count (%)	Mean (SD) or count (%)	
**Toenail arsenic (µg/g)**	1.02 (1.6)	1.11 (2.0)	n.s.
**Plasma folate (ng/mL)**	8.38 (5.3)	7.46 (4.4)	n.s.
**Plasma folate (binned)**			n.s.
**Insufficient (< 4 ng/mL)**	59 (24.0%)	32 (25.0%)	
**Sufficient (≥ 4 ng/mL)**	187 (76.0%)	96 (75.0%)	
**Maternal age (years)**	24.5 (4.5)	24.2 (4.8)	n.s.
**Maternal BMI**	23.71 (4.1)	23.05 (4.5)	n.s.
**Maternal education (< university)**	233 (94.7%)	112 (87.5%)	0.023
**Child sex (female)**	121 (49.2%)	45 (35.2%)	0.013
**Child age (days)**	75.44 (85.2)	187.23 (97.4)	< 0.001
**Child level of spinal defect**			
Lumbar/lumbosacral/sacral	238 (96.7)	N/A	
Cervical/thoracic	8 (3.3)	N/A	
**Pre/peri-natal folic acid supplementation (yes)**	41 (16.7%)	33 (25.8%)	n.s.
**Plasma vitamin B12 (pg/mL)**	375.34 (155.1)	407.86 (153.7)	n.s.
**Estimated cell type proportions**			
B cells	0.06 (0.02)	0.06 (0.02)	n.s.
CD4T	0.14 (0.04)	0.15 (0.04)	n.s.
CD8T	0.12 (0.05)	0.11 (0.04)	n.s.
Monocytes	0.06 (0.02)	0.06 (0.03)	n.s.
Natural killer cells	0.05 (0.02)	0.05 (0.02)	0.013
Neutrophils	0.57 (0.11)	0.56 (0.11)	n.s.

*p values were obtained from t-tests for continuous variables, and Chi-square tests for categorical variables (Fisher’s exact tests were used where cell counts were less than 5)

### Data characterization

Principal components analysis was conducted on the processed and batch-corrected DNAme data to investigate primary variables (biological and technical) associated with DNAme variation. The top ten principal components (PCs) explained 26.8% of the DNAme variance and were primarily associated with cell composition (PCs 1–4), Supplementary Fig. 2. Plasma folate was associated with PC3 (1.2% of the variance, *R* = −0.13, *p* < 0.05) and with PCs 8 and 9, which explained 0.7% and 0.6% of the variance, respectively. Spina bifida status and arsenic were not associated with the top 10 PCs. EPIC array row (1–8) was associated with PCs 4, 5, and 7–9.

### Epigenome-wide association studies

**Fig. 2:**
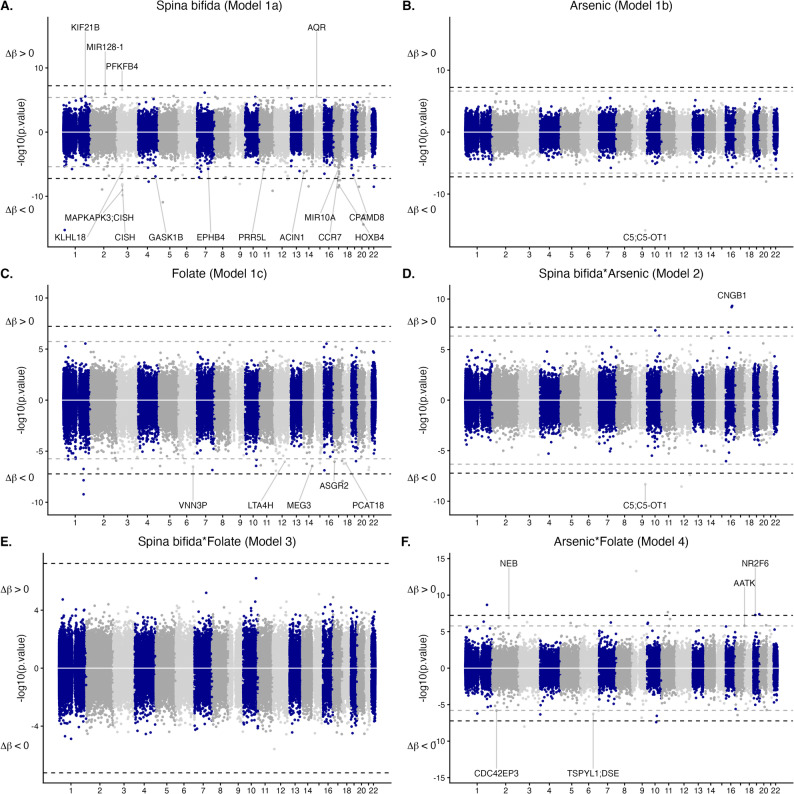
Miami plots of epigenome-wide linear modelling results. For all plots, the -log10 of the unadjusted p values are shown on the Y axis (positive DNAme effect directions shown > 0, negative effect directions shown < 0), hg38 chromosome position is plotted along the X axis. Horizontal dashed grey intercepts delineate genome-wide significance at 9E-8, any genes overlapping CpGs that reach genome-wide significance are labelled, overlapping transcripts are separated by semi colons. **A** Spina bifida status, Model 1a. **B** Main effect of toenail arsenic (µg/g), Model 1b. **C** Main effect of plasma folate (ng/mL), Model 1c. **D** Interaction of spina bifida status and toenail arsenic (µg/g), Model 2. **E** Interaction of spina bifida status and plasma folate (ng/mL), Model 3. **F** Interaction of toenail arsenic (µg/g) and plasma folate (ng/mL), Model 4

A summary of results for all epigenome-wide association studies (EWAS) is shown in Table 2. The differentially methylated CpGs reaching Bonferroni and FDR significance in each EWAS are shown in Table 3, all CpGs reaching FDR significance along with model estimates and genomic annotation are shown in Supplementary Table 1. Manhattan plots are shown in Fig. 2, Q-Q plots and genomic inflation factors (λ) are shown in Supplementary Fig. 3. All models included main terms for spina bifida, arsenic, and folate, as well as maternal age (years), BMI, education (as a proxy for socioeconomic status), EPIC array row, and the six estimated blood cell proportions.

**Table 2:** Summary of results from epigenome-wide association studies conducted as part of the analysis. DMR indicates differentially methylated region

Model	Variable of interest	Genomic inflationλ	CpGs FDR < 0.05	CpGs Bonferroni *p* < 0.05	DMRs FDR < 0.05
1a	Spina bifida	1.10	71	22	9
1b	Arsenic	0.92	6	4	1
1c	Folate	1.02	33	3	5
2	Spina bifida*Arsenic	0.86	11	6	8
3	Spina bifida*Folate	0.97	0	0	0
4	Arsenic*Folate	0.88	28	7	25

### Spina bifida main effect

**Fig. 3:**
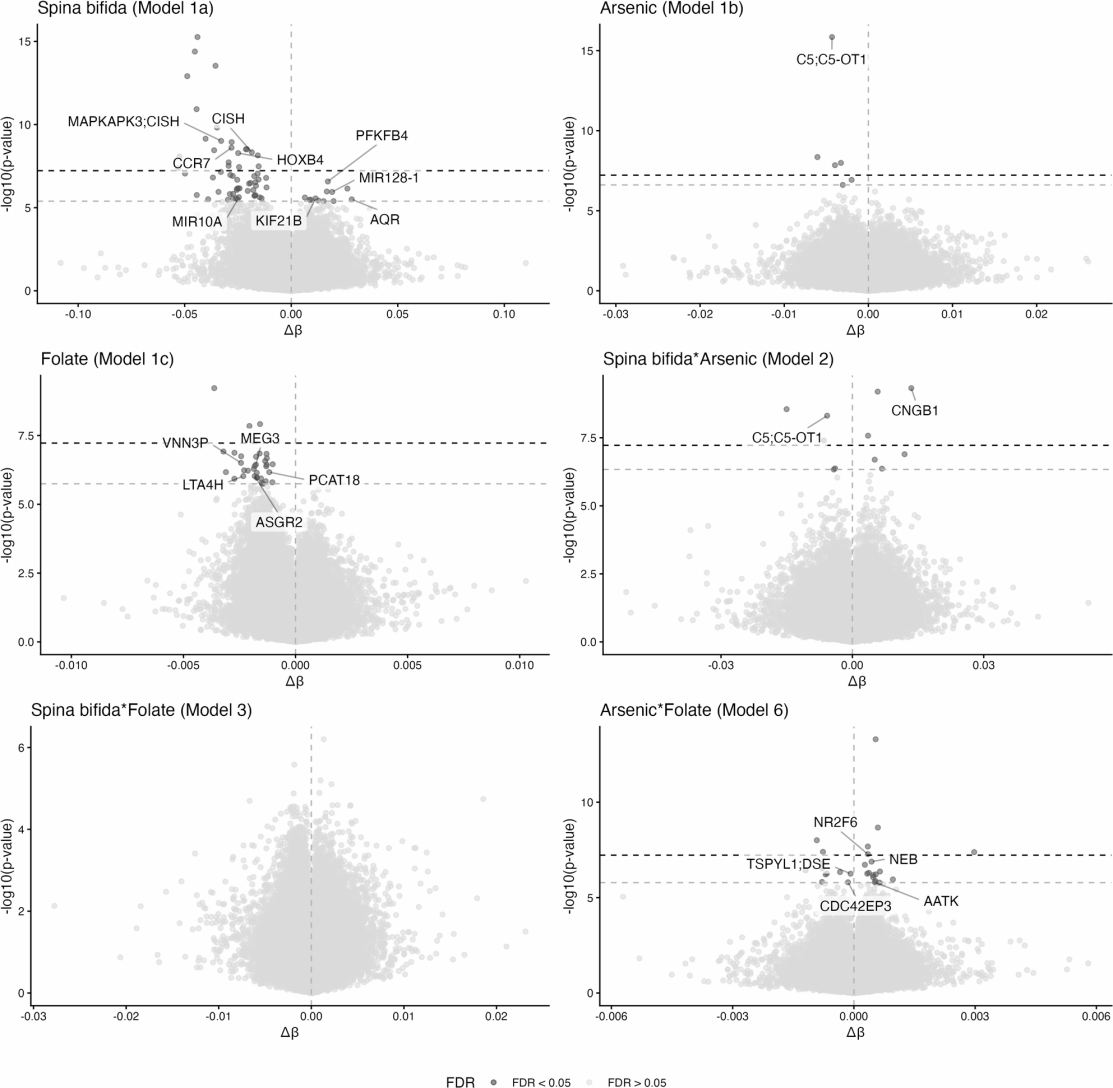
Volcano plots of epigenome-wide linear modelling results. For all plots, the -log10 of the nominal p value is shown on the Y axis, with the effect size per unit change of the covariate of interest shown along the X axis (Δβ). For spina bifida status models, control individuals were used as the referent group. Each point represents a CpG, and CpGs meeting FDR < 0.05 for each model are indicated in dark grey. A vertical dashed intercept indicates Δβ = 0. CpGs with FDR < 0.05 are labelled with any associated gene names, overlapping transcripts are separated by semi colons. **(A)** Spina bifida status, Model (1) **(B)** Interaction of spina bifida status and toenail arsenic (µg/g), Model (2) **(C)** Interaction of spina bifida status and plasma folate (ng/mL), Model (3) **(D)** Main effect of toenail arsenic (µg/g), Model (4) **(E)** Main effect of plasma folate (ng/mL), Model (5) **(F)** Interaction of toenail arsenic (µg/g) and plasma folate (ng/mL), Model 6

EWAS Model 1a investigated the main effect of spina bifida (λ = 1.10) with 71 CpGs reaching FDR significance and 22 reaching Bonferroni *p* < 0.05, Table 2. The control samples (mothers of children without spina bifida) were treated as the referent group in this analysis, and the majority of FDR-significant CpGs (*n* = 58, 81.6%) were inversely associated with DNAme, i.e. mothers of spina-bifida-affected offspring had lower mean DNAme at the majority of differentially methylated loci as compared to controls, Fig. 3A. Consideration of DNAme effect sizes (in β value units [56]) demonstrated that more than half of FDR significant CpGs had Δβ > |0.02| (43 of 71 CpGs, 61%), two of which had Δβ > |0.05| (both in the negative direction). The CpGs with the largest effect sizes were cg00052692 (chr12:122,004,957, Δβ = −0.053) and cg13887723 (chr9:88,529,984, Δβ = −0.052). These CpGs both reached Bonferroni significance; both were intergenic and did not overlap any UCSC reference genes, functional regions, or CpG islands. The cg00052692 locus was adjacent to another CpG (cg04753874, chr12:122,004,950); together, these two CpGs were detected as a spina bifida-associated DMR.

Considering all 71 FDR-significant CpGs associated with spina bifida, 13 contributed to five larger differentially methylated regions (DMRs) associated with spina bifida, which overlapped the *MAPKAP3* gene (4 CpGs, chr3:50,612,563 − 50,613,332), *HOXB3* (13 CpGs, chr17:48,579,181 − 48,580,808), and *CCR7* (4 CpGs, chr17:40,560,550 − 40,561,041), Supplementary Table 2. There were four additional DMRs associated with spina bifida that did not overlap CpGs from the EWAS Model 1a. Two of these DMRs occurred on chromosome 17, overlapping *VMP1* (4 CpGs, chr17:59,838,304 − 59,838,412), and *MCRIP1* (21 CpGs, chr17:81,821,352 − 81,822,448); the other two overlapped *RUNX3* (14 CpGs, chr1:24,964,894 − 24,965,581) and *IL32* (6 CpGs, chr16:3,065,551-3,065,808). All DMRs associated with spina bifida had lower mean DNAme in mothers of spina bifida cases as compared to controls.

**Table 3:** Top significant results of epigenome-wide association studies. For brevity this table indicates CpGs associated at Bonferroni significance with spina bifida case/control status (Model 1a), arsenic (Model 1b), folate (Model 1c), the interaction of spina bifida and arsenic (Model 2), the interaction of spina bifida and folate (Model 3), or the interaction of arsenic and folate (Model 4). For an expanded list of all CpGs meeting FDR significance see Supplementary table 1. Gene and genomic region annotations were sourced from the Illumina v2.0 manifest

Probe ID	Chr	Position (hg38)	Mean β (all samples)	Estimate (M values)	Estimate (β values)	*p*-value	Gene(s)	Genomic region(s)
Spina bifida main effect (Model 1a)								
cg06072257	chr1	11,374,579	0.72	−0.304	−0.044	5.46E-16		
cg00163095	chr2	106,961,010	0.40	−0.170	−0.028	1.14E-09		
cg04901989	chr3	50,605,514	0.50	−0.091	−0.016	7.15E-09		
cg05541267	chr3	50,609,943	0.71	−0.240	−0.035	1.52E-10		
cg08996521	chr3	50,612,563	0.15	−0.225	−0.018	4.64E-09	*CISH; CISH*	TSS1500; TSS1500
cg05186879	chr3	50,612,891	0.58	−0.193	−0.033	9.60E-10	*MAPKAPK3; MAPKAPK3; CISH; CISH*	5UTR; exon_2; TSS1500; TSS1500
cg18821474	chr4	95,233,024	0.41	−0.177	−0.029	1.89E-08		
cg04213565	chr5	35,899,937	0.26	−0.363	−0.044	1.19E-11		
cg08712677	chr5	151,078,927	0.33	−0.164	−0.024	3.61E-08		
cg13887723	chr9	88,529,984	0.48	−0.305	−0.052	8.43E-09		
cg15885703	chr11	118,224,115	0.50	−0.232	−0.040	7.16E-10		
cg00052692	chr12	122,004,957	0.46	−0.312	−0.053	4.31E-08		
cg19627960	chr14	67,480,205	0.65	−0.226	−0.036	3.51E-09		
cg07248223	chr17	40,561,023	0.47	−0.163	−0.028	2.48E-09	*CCR7; CCR7; CCR7*	TSS1500; TSS200; TSS200
cg22525159	chr17	40,561,041	0.27	−0.227	−0.029	3.05E-08	*CCR7; CCR7; CCR7*	TSS1500; TSS200; TSS200
cg05822888	chr17	48,579,530	0.36	−0.159	−0.025	5.22E-09	*HOXB4*	TSS1500
cg20918823	chr17	56,174,100	0.13	−0.207	−0.015	3.27E-08		
cg08076236	chr20	10,662,651	0.58	−0.126	−0.021	3.06E-09		
cg25377444	chr20	50,284,883	0.51	−0.261	−0.045	4.07E-15		
cg25377445	chr20	50,284,895	0.81	−0.319	−0.036	2.93E-14		
cg25499898	chr20	60,063,115	0.76	−0.372	−0.049	1.23E-13		
cg26284113	chr22	36,197,981	0.43	−0.123	−0.021	3.07E-09		
**Arsenic main effect (Model 1b)**								
cg09454603	chr6	38,220,201	0.88	−0.084	−0.006	4.44E-09		
cg20363688	chr9	120,953,806	0.92	−0.089	−0.004	1.41E-16	*C5; C5; C5-OT1*	exon_40; exon_40; TSS1500
cg17777559	chr11	17,579,889	0.88	−0.054	−0.004	1.43E-08		
cg14876896	chr20	58,373,161	0.92	−0.064	−0.003	1.02E-08		
**Folate main effect (Model 1c)**								
cg01711159	chr1	185,369,263	0.62	−0.013	−0.002	1.43E-08		
cg24854225	chr1	185,405,402	0.75	−0.028	−0.004	6.03E-10		
cg11153071	chr17	80,774,277	0.56	−0.009	−0.002	1.21E-08		
Spina bifida*Arsenic interaction effect (Model 2)								
cg03039127	chr3	100,404,312	0.92	0.074	0.004	2.64E-08		
cg20363688	chr9	120,953,806	0.92	−0.118	−0.006	4.87E-09	*C5; C5; C5-OT1*	exon_40; exon_40; TSS1500
cg17562253	chr12	47,903,846	0.78	−0.122	−0.015	2.81E-09		
cg18292079	chr12	125,123,582	0.88	−0.088	−0.007	3.96E-08		
cg21502657	chr16	52,609,219	0.91	0.111	0.006	6.35E-10		
cg04269043	chr16	57,884,139	0.45	0.078	0.013	4.74E-10	*CNGB1; CNGB1; CNGB1; CNGB1*	3UTR; exon_33; 3UTR; exon_33
Spina bifida*Folate interaction effect (Model 3)								
No results at Bonferroni *p* < 0.05								
Arsenic*Folate interaction effect (Model 4)								
cg01790787	chr1	199,081,356	0.88	0.008	0.001	2.14E-09		
cg18152055	chr3	51,878,511	0.88	−0.012	−0.001	9.87E-09		
cg13627584	chr9	37,853,488	0.94	0.014	0.001	5.06E-14		
cg14112772	chr10	83,425,300	0.77	−0.006	−0.001	4.01E-08		
cg16363615	chr11	57,434,069	0.95	0.010	0.000	2.10E-08		
cg14989308	chr19	17,246,663	0.05	0.010	0.000	5.28E-08	*NR2F6*	TSS1500
cg06599908	chr19	54,420,458	0.52	0.017	0.003	4.13E-08		

### Arsenic main effect

EWAS Model 1b investigated the main effect of maternal toenail arsenic on leukocyte DNAme (λ = 0.92) and resulted in six CpGs reaching FDR significance, four of which also met Bonferroni significance. All six CpGs were inversely associated with arsenic, meaning that mean DNAme decreased with increasing arsenic concentrations, Fig. 3B. These CpGs were largely intergenic, except for one CpG overlapping the gene body of *C5* on chromosome 9 (chr9:120,953,806). None of the FDR-significant CpGs associated with arsenic were part of larger DMRs, although one DMR was identified in association with arsenic, spanning six CpGs on chromosome 2 (chr2:27,307,802 − 27,308,302) and overlapping the gene body of *UCN*, Supplementary Table 2. Contrary to the individually significant CpGs associated with arsenic, this DMR was positively associated with arsenic concentration (higher DNAme in individuals with higher mean toenail arsenic).

### Folate main effect

EWAS Model 1c evaluated DNAme associated with plasma folate (λ = 1.02), with 33 CpGs reaching FDR significance, three of which met Bonferroni significance. Similar to what was observed for toenail arsenic (Model 1b), all CpGs associated with plasma folate at FDR and Bonferroni significance showed inverse associations with mean DNAme levels decreasing with increasing folate levels, Fig. 3C. The three folate-associated CpGs at Bonferroni significance were intergenic, while those that met FDR significance overlapped *VNN3P* (chr6:132,734,486), the maternally expressed imprinted gene *MEG3* (chr14:100,848,018), *PCAT18* (chr18:26,703,411), *LTA4H* (chr12:96,044,806), and *ASGR2* (chr17:7,115,943).

Three of the folate-associated CpGs were part of five larger folate DMRs. Two of the folate DMRs were located on chromosome 12, one intergenic comprised 2 CpGs (chr12:14,260,647 − 14,260,756), the other comprised 8 CpGs and overlapped both *AC008012.1* and *FGF23* (chr12:4,379,583-4,380,055); while the third folate DMR was located on chromosome 17 and overlapped the *RPTOR* gene (5 CpGs, chr17:80,774,134 − 80,774,433). Two additional folate DMRs did not overlap FDR-significant CpGs, but were located on chromosome 1 overlapping *CHI2L2* and *DENN2D* (10 CpGs, chr1:111,200,292 − 111,200,915) and on chromosome 6 overlapping *SLC28A8* (chr6:36,024,723 − 36,024,914). As with the folate-associated CpGs, all folate-associated DMRs were inversely associated with plasma folate.

### Effect modification of spina bifida by arsenic

We next sought to evaluate effect modification of spina bifida status by arsenic (Model 2), spina bifida status by folate (Model 3), and arsenic by folate (Model 4), by adding these three cross-product interaction terms one at a time to the marginal model described above. The interaction of arsenic and spina bifida (Model 2) was pursued to identify CpGs where the quantitative association between arsenic and maternal DNAme differed significantly between mothers of cases versus mothers of controls, indicating potential differential sensitivity to arsenic. Similarly, the interaction between folate and spina bifida (Model 3) was run to identify loci where the relationship between folate and the maternal epigenome was altered between case and control mothers, thus highlighting regions with potential relevance to the protective effect of folate status. Finally, the interaction between arsenic and folate (Model 4) was investigated to identify CpGs labile to both, the existence of which would support the hypothesis of molecular crosstalk involving OCM. Acknowledging the size of the study sample, these interaction models were run as exploratory analyses and interpreted cautiously.

The interaction of spina bifida status and arsenic (Model 2) was associated with differential DNAme at 11 CpGs (FDR < 0.05), six of which met Bonferroni significance. Folate did not significantly interact with spina bifida at any loci (Model 3). The interaction of arsenic and folate (Model 4) was associated with DNAme at 28 CpGs (FDR < 0.05), seven of which met Bonferroni significance. FDR-significant results for all interaction models are presented in Supplementary Table 1.

To characterize the effect modification of arsenic on spina bifida status (Model 2), we evaluated the impact of arsenic on DNAme at the 11 CpGs that met FDR significance in the interaction model. Stratified analyses were conducted separately to test for the main effect of arsenic in the spina bifida cases (*n* = 246) and controls (*n* = 128); all 11 CpGs reached nominal significance in one or both strata, Table 4. Among these 11 CpGs, three (cg11259443, cg20363688, cg18292079) were significantly associated with arsenic only among spina bifida cases, all of which showed decreasing DNAme with increasing arsenic levels. Among these three CpGs, cg20363688 localizes to the gene body of *C5*. Four CpGs (cg03039127, cg04741292, cg16947025, cg04269043) were significantly associated with arsenic only among controls, all of which also showed decreasing DNAme in response to arsenic. Of these CpGs, cg04269043 maps to the *CNGB1* gene, which encodes a subunit for cation channel proteins, and in which loss of function mutations are pathogenic for a subtype of autosomal recessive retinitis pigmentosa [14]. The final four CpGs (cg17562253, cg05395476, cg21502657, cg25185444) were significantly associated with arsenic in both cases and controls and showed opposing directions of effect in the two groups. Taken together, these stratified results suggest a complex and often opposing DNAme response to arsenic, one which may differ between spina bifida (case) and control mothers.

**Table 4:** Evaluation of the 11 CpGs associated with the interaction of spina bifida status and arsenic at FDR < 0.05 (Model 2). Estimates (M value scale) and p values from linear models evaluating an arsenic main effect in stratified spina bifida cases (*n* = 246) and controls (*n* = 128) are presented. Genes were annotated using the Illumina v2.0 manifest

				Spina bifida birth (*N* = 246)		Non-spina bifida birth (control) (*N* = 128)				
Probe ID	Chr	Pos (hg38)	Mean β (all samples)	Estimate (95% CI)	*p*-value	Estimate (95% CI)	*p*-value	Gene(s)	Significance	Direction
cg11259443	chr2	11,628,485	0.92	−0.075	7.70E-09	0.010	0.32		SB	Neg in SB
cg03039127	chr3	100,404,312	0.92	0.005	0.490	−0.070	3.27E-07		Control	Neg in Control
cg20363688	chr9	120,953,806	0.92	−0.144	9.77E-21	−0.024	0.10	*C5; C5; C5-OT1*	SB	Neg in SB
cg04741292	chr10	75,373,792	0.88	0.007	0.732	−0.172	2.00E-07		Control	Neg in Control
cg16947025	chr10	110,358,930	0.82	0.015	0.096	−0.055	2.43E-06		Control	Neg in Control
cg17562253	chr12	47,903,846	0.78	−0.087	2.04E-08	0.036	5E-3		Both	Opposing
cg18292079	chr12	125,123,582	0.88	−0.075	1.46E-10	0.016	0.15		SB	Neg in SB
cg05395476	chr16	21,155,244	0.91	0.029	0.006	−0.071	2.80E-05		Both	Opposing
cg21502657	chr16	52,609,219	0.91	0.024	0.036	−0.087	1.17E-07		Both	Opposing
cg04269043	chr16	57,884,139	0.45	0.004	0.439	−0.074	3.51E-07	*CNGB1; CNGB1; CNGB1; CNGB1*	Control	Neg in Control
cg25185444	chr20	34,528,898	0.93	−0.052	1.87E-05	0.029	0.01		Both	Opposing

### Effect modification of arsenic by folate

We similarly sought to characterize the interaction of arsenic and folate by conducting stratified analyses for a main effect of arsenic in individuals with low (< 4 ng/mL) or high (≥ 4 ng/mL) plasma folate, Table 5. The 4 ng/mL threshold was chosen such that the low folate group reflects clinical folate insufficiency [57]. At the 28 CpGs associated with the interaction of arsenic and folate, all but two CpGs were nominally associated with arsenic in one or both folate strata (*n* = 26). Half of the CpGs (*n* = 14) were significantly associated with arsenic only in the low folate group; 12 exhibited decreasing DNAme with increasing arsenic, including cg07649828 in the gene body of *NEB*, and cg15131155 in the gene body of *AATK*. The remaining were intergenic (cg01579603, cg05145011, cg06599908, cg07649828, cg13627584, cg15131155, cg15686265, cg16190209, cg16657741, cg20140577, cg25013235, cg25696631). The other 2/14 CpGs showed increasing mean DNAme with increasing arsenic (cg14420589 in *TSPYL1* and *DSE*, and cg21914984 in *CDC42EP053*). Of the remaining 12 CpGs associated with the interaction of arsenic and folate half (*n* = 6) were associated with arsenic only in the high-folate group and showed decreasing DNAme with increasing arsenic (cg08558975, cg12459844, cg13876641, cg14112772, cg18152055, cg24186681). The other 6 CpGs were associated with arsenic in both low and high folate groups. Taken together the results of these folate-stratified arsenic models suggest that arsenic-associated DNAme changes may be more pronounced in low-folate individuals.

**Table 5:** Evaluation of the 28 CpGs associated with the interaction of arsenic and folate at FDR < 0.05 (Model 4). Estimates (M value scale) and p values from linear models evaluating an arsenic main effect in high folate (≥ 4 ng/mL, *n* = 91) and low folate (< 4 ng/mL, *n* = 283) strata

				Folate < 4 ng/mL (*N* = 91)		Folate ≥ 4 ng/mL (*N* = 283)				
Probe ID	Chr	Position (hg38)	Mean β (all samples)	Estimate (95% CI)	*p*-value	Estimate (95% CI)	*p*-value	Gene(s)	Significance	Direction
cg25320780	chr1	110,390,723	0.87	0.011	0.46	−0.021	0.06		Neither	-
cg01579603	chr1	168,499,899	0.84	−0.083	5.2E-07	0.000	0.97		Low	Neg in Low Folate
cg01790787	chr1	199,081,356	0.88	−0.098	4.5E-09	0.016	0.03		Both	Opposing
cg21914984	chr2	37,672,321	0.02	0.180	9.1E-13	0.008	0.51	*CDC42EP3;CDC42EP3;CDC42EP3;CDC42EP3*	Low	Pos in Low Folate
cg07649828	chr2	151,735,201	0.90	−0.096	1.8E-09	0.005	0.45	*NEB; NEB; NEB; NEB*	Low	Neg in Low Folate
cg18152055	chr3	51,878,511	0.88	0.020	0.41	−0.050	4.20E-05		High	Neg in High Folate
cg05145011	chr3	78,139,564	0.91	−0.059	2.0E-05	−0.006	0.46		Low	Neg in Low Folate
cg26522415	chr4	925,898	0.93	0.033	0.017	−0.023	0.02		Both	Opposing
cg20140577	chr5	90,357,536	0.89	−0.083	7.2E-06	0.002	0.79		Low	Neg in Low Folate
cg12459844	chr5	135,127,344	0.79	0.016	0.089	−0.019	0.006		High	Neg in High Folate
cg09454603	chr6	38,220,201	0.88	−0.156	2.1E-09	−0.055	0.001		Both	Neg in Both
cg14420589	chr6	116,279,978	0.03	0.053	1.9E-06	0.003	0.48	*TSPYL1;DSE; DSE; DSE; DSE; DSE*	Low	Pos in Low Folate
cg00386035	chr6	165,825,218	0.68	0.019	0.044	−0.015	0.022		Both	Opposing
cg16190209	chr7	108,034,016	0.94	−0.139	1.2E-09	−0.003	0.67		Low	Neg in Low Folate
cg13627584	chr9	37,853,488	0.94	−0.184	4.1E-16	0.004	0.64		Low	Neg in Low Folate
cg13876641	chr9	87,662,837	0.87	0.015	0.38	−0.042	2.7E-04		High	Neg in High Folate
cg15686265	chr10	3,924,390	0.93	−0.131	6.7E-08	0.002	0.85		Low	Neg in Low Folate
cg25013235	chr10	7,593,226	0.89	−0.085	2.3E-05	0.002	0.74		Low	Neg in Low Folate
cg14112772	chr10	83,425,300	0.77	0.015	0.17	−0.025	3.0E-04		High	Neg in High Folate
cg08558975	chr10	88,865,537	0.89	0.029	0.15	−0.055	6.8E-05		High	Neg in High Folate
cg16363615	chr11	57,434,069	0.95	−0.128	4.0E-08	0.019	0.043		Both	Opposing
cg25696631	chr11	65,072,780	0.79	−0.081	6.8E-09	0.007	0.21		Low	Neg in Low Folate
cg16657741	chr11	77,337,237	0.95	−0.121	9.3E-10	0.002	0.85		Low	Neg in Low Folate
cg24186681	chr17	17,248,062	0.79	0.018	0.32	−0.038	0.002		High	Neg in High Folate
cg15131155	chr17	81,132,099	0.92	−0.149	7.2E-09	0.007	0.52	*AATK*	Low	Neg in Low Folate
cg14989308	chr19	17,246,663	0.05	−0.024	0.16	0.022	0.059	*NR2F6*	Neither	-
cg06599908	chr19	54,420,458	0.52	−0.203	1.6E-08	0.018	0.28		Low	Neg in Low Folate
cg14876896	chr20	58,373,161	0.92	−0.150	2.2E-09	−0.031	0.013		Both	Neg in Both

### Gene set enrichment analysis

The CpGs that reached FDR < 0.05 in each EWAS model were submitted in separate queries for gene set enrichment analysis against the GO and KEGG databases. At FDR < 0.05, no GO or KEGG terms were significantly enriched in the results of any of the six models. Considering nominal p-values < 0.01, Model 1a yielded five GO biological process terms, all of which were associated with the same two genes (*CCR7*, *CISH*). The five terms related to cytokine and immune-signaling terms (response to cytokine, cytokine-mediated signaling, cellular response to cytokine stimulus, response to peptide, G protein-coupled receptor signaling pathway). These maternal leukocyte DNAme GO terms may suggest inflammatory/immune differences associated with a prior spina bifida pregnancy, though future work is required to confirm these findings.

### Evaluation of previously identified differentially methylated CpGs

To contextualize our significant findings in the existing literature and validate the robustness of our dataset we performed a look-up analysis. First, we evaluated whether the CpGs that reached FDR significance in our main effects analysis had been reported in curated lists from prior EWAS of spina bifida [60], arsenic exposure [61–64], and folate [65–67], this analysis revealed no overlap. We subsequently expanded this look-up to CpGs reaching nominal significance (*p* < 0.05) in our models: 2, 24, and 28 CpGs were previously reported to be associated with spina bifida, arsenic, and folate, respectively (Supplementary Table 2).

Considering the identity of the previously-reported CpGs, our maternal DNAme data replicated two of 77 spina bifida CpGs from a study of leukocyte DNAme in adult patients by Rochtus et al. [60], both with a consistent direction of effect between studies (lower DNAme in spina bifida versus controls). These CpGs were in the TSS1500s of the *C10orf120* (cg15840151) and *YPEL4* (cg03773647). Twenty four CpGs had been previously associated with arsenic, including 12 reported by Demanelis et al. [64] in a study of urinary arsenic in the Health Effects of Arsenic Longitudinal Study (HEALS) cohort from Bangladesh, ten of which went in a consistent direction in our study as originally reported, including the study’s top hit upstream of *ABR* (cg10003262). Similarly, six CpGs at p < 0.05 were previously reported in association with arsenic in a Bangladeshi cohort by Argos et al. (2015); all went in the same direction as originally reported. Five CpGs associated with toenail arsenic in Wang et al. [63] reached significance in our cohort (cg01167323, cg25545401, cg16321846, cg07151197, cg07441152), three of which went in the same direction (inverse) in our study, including CpGs in *PLEKHG4* and *SDK1*. One CpG reportedz to be associated with arsenic exposure by Bozack et al. [68] reached nominal significance in our study (cg18616702 on chr1 in the 5’ untranslated region of *ATAMTSL4* and the TSS1500 of *MIR4257*), although our finding went in the opposite direction as originally reported. For folate, we observed notable overlap with the seminal cord blood meta-analysis by Joubert et al. (2016), where 28 of 443 CpGs (6.1%) reached nominal significance (*p* < 0.05) in our data; all had consistent DNAme effects between studies (all lower DNAme with increasing folate). We did not observe replication of hits from studies of maternal serum folate or dietary intake [65, 66].

## Discussion

In this study we assessed maternal leukocyte DNAme patterns associated with spina bifida, toenail arsenic, and serum folate in a case-control cohort of Bangladeshi women with and without a previous spina bifida-affected birth. At FDR significance, 71 CpGs were associated with offspring spina bifida status, 6 with toenail arsenic, and 33 with serum folate. Interactions were detected for spina bifida*arsenic (11 CpGs) and arsenic*folate (28 CpGs), no interactions were observed for spina bifida*folate. Overall, our findings suggest that offspring spina bifida is associated with a maternal leukocyte DNAme signature, which is modified by chronic arsenic exposure at several loci. Arsenic and folate show distinct non-overlapping leukocyte DNAme signatures; however, at loci sensitive to both, arsenic effects were generally more pronounced in individuals with low serum folate.

While prior work has evaluated DNAme signatures in NTD-affected infants and adults [60, 69, 70], here, we evaluate maternal leukocyte DNAme relative to offspring spina bifida. Among spina bifida-associated loci, we detected two differentially methylated Homeobox (HOX) genes, a family of evolutionarily conserved and developmentally important transcription factors [71]. First, we detected a CpG upstream of *HOXB4*, which showed lower mean DNAme in mothers of cases, and also observed two DMRs in *HOXB3* associated with offspring spina bifida and the interaction of spina bifida*arsenic, respectively. Our previous study of dural tissue from affected infants reported arsenic-associated DNAme at in *HOCX4*, a paralog of *HOXB4*; both *HOXB4* and *HOXC4* are upregulated in retinoic acid-induced mouse models of NTDs [72], suggesting possible convergent effects of spina bifida and arsenic at HOX genes. We also identified CpGs in genes previously implicated in NTD biology including *PFKFB4* (involved in neural folding, embryonic depletion results in craniofacial defects) [73], and *MIR10A* (upregulated in retinoic-acid induced NTDs) [74]. Altered DNAme at developmentally important genes in maternal tissue may reflect an underlying genomic signature related to NTD risk, or a persistent postnatal impact of gestational spina bifida exposure.

Most loci associated with offspring spina bifida showed lower mean DNAme in mothers of cases relative to controls, consistent with previous literature reports. For example, early work using an antibody-based global DNAme assay detected lower average DNAme genome-wide in 65 NTD-affected fetal brain samples relative to age-matched controls [41], and similar work found that both global DNAme and DNAme at long-interspersed nucleotide elements (LINE-1 repeats) were lower in NTD-affected fetal neural tissue versus controls [42]. Further, in an epigenome-wide association study of adults with myelomeningocele, most significant CpGs had lower mean DNAme in cases versus controls [60]. Our findings are the first to report this pattern in maternal blood, possibly reflecting maternal factors influencing spina bifida-associated DNAme.

We observed significant effect modification of spina bifida-associated DNAme by maternal arsenic. One CpG in *C5* (complement 5) was associated with both an inverse main effect of arsenic and a significant spina bifida*arsenic interaction. Stratified analyses indicated that arsenic was associated with this CpG only in mothers of cases. This CpG may act as a biomarker of arsenic sensitivity, or may indicate that a proportion of the DNAme signature associated with spina bifida at this locus depends on environmental arsenic status, aligning with previous hypotheses [75]. *C5* is part of the innate immune system, although recent work suggests the complement system also has many non-immune functions; for example, *C5* expression has been detected in the epithelium of the developing neural tube of both mice and humans [76], and restriction of *C5* signaling in folate-deficient mice yields NTD-associated malformations including anencephaly and exencephaly [76]. These results may indicate a role for *C5* in the pathogenesis of spina bifida.

We detected no CpGs significantly associated with the interaction of offspring spina bifida and maternal serum folate, suggesting folate effects on leukocyte DNAme are largely consistent across this study sample regardless of spina bifida birth history. This result is perhaps surprising, given the known impacts of maternal folate status on spina bifida risk and the fact that both offspring spina bifida status and serum folate levels were associated with DNAme effects in our cohort. It is possible that folate-associated DNAme signatures would be more apparent perinatally, or that we are underpowered to detect more subtle differences with the current sample size. Ultimately, we observe that the high-level effects of folate are consistent across individuals with different reproductive histories, aligning with recommendations for folic acid supplementation regardless of pre-ascertained NTD risk.

Paralleling the directional pattern observed at CpGs associated with offspring spina bifida, higher toenail arsenic and serum folate levels were each associated with reduced mean DNAme at FDR-significant CpGs. Literature precedent supports decreasing cord blood DNAme with increasing maternal folate, including at 95% of the loci associated with prenatal maternal folate in Joubert et al. (2016) [67]. Reduced DNAme with increased folic acid intake (food frequency questionnaires) has also been observed in adult blood [66, 77] and neonatal blood [78]. It has been postulated elsewhere that folate may exert negative feedback on methylation processes, leading to reduced DNAme as folate availability increases, possibly explaining this directional association [78]. By contrast, the exclusively inverse arsenic associations we observe are somewhat novel and may vary by tissue or method of arsenic assessment. Our results mirror the results of Wang et al. [63] studying toenail arsenic in elderly white American men, in which 69/72 CpGs (96%) had decreasing DNAme per increase in arsenic interquartile range. Similarly, in leukocyte DNAme, Demanelis et al. [64] reported a majority of inverse associations (68%) with urinary arsenic in the HEALS cohort. However, in other work the majority of significant buccal and blood DNAme were positively associated with urinary arsenic in American Indians [68] and Argentinian women [79]. In whole blood, Argos et al. [62] reported 16/35 CpGs inversely associated with arsenic (measured in blood and urine) in a cohort from rural Bangladesh. Compared with urine-based arsenic measurements, toenail arsenic measurements reflect the prior 5–18 months of exposure and may favor detection of inorganic arsenic [80]. In general, prior literature supports decreasing DNAme with increasing toenail arsenic, and between-study variability likely reflects variation in tissue, targeted arsenic metabolite, and study population. These effects should be disentangled going forward to better characterize arsenic-associated DNAme variation.

Folate was uniquely associated with a CpG in the *MEG3* gene body (cg23722168). *MEG3* is a maternally expressed imprinted gene, and higher plasma folate was associated with lower mean DNAme at this locus. Of note, the differentially methylated CpG lies outside of the *MEG3* imprinting control centers, both of which occur upstream of the transcription start site [81]. Although the functional implications of differential DNAme with folate at this locus are unclear, previous work has related prenatal maternal folate to DNAme at other imprinted loci including *H19* (lower cord blood DNAme with higher reported prenatal folic acid) [82] and the imprinting regulator *ZFP57* (lower DNAme in cultured cord blood immune cells exposed to high folate) [83]. Our work thus adds *MEG3* to the list of imprinted genes with folate-responsive DNAme.

In this study we observed that arsenic and folate interacted with DNAme at 28 CpGs. In stratified analyses, half of these loci associated with arsenic only among individuals with low serum folate (< 4 ng/mL), despite this low folate group being smaller and less statistically powered; most of these loci showed inverse associations. These results together indicate that low serum folate may increase epigenetic sensitivity to arsenic and reinforces the observed pattern of decreased DNAme with increasing arsenic.

In the context of previous literature, we replicated 28 of 443 folate-associated CpGs reported in cord blood by Joubert et al. (2016). Although modest, this replication suggests folate sensitivity at these loci across both tissues and the life course. Genes overlapping these cross-tissue folate CpGs include *CCDC177*, *CHRD*, *CPLX1*, *NPAS4*, *OSBPL10*, *PCP2*, and *TMEM200C*. Absent clear reports linking these genes to folate in other contexts, our results suggest that these genes may be worth considering in future studies of folate-associated DNAme.

Strengths of this study include direct measurements of toenail arsenic and serum folate, improving exposure precision over geospatial estimation of arsenic exposure, or dietary questionnaires of folic acid intake. Additionally, toenail arsenic reflecting the past 5–18 months of arsenic exposure [80] appropriately captures the perinatal window in our postnatal cohort. An additional strength of this cohort is the sample size and clinical characterization relative to the phenotype rarity. Additionally, while several studies investigate spina bifida or NTD index patients, investigating maternal tissue in this disease context is novel. Limitations include postnatal blood sampling for DNAme, complicating the interpretation of folate and spina bifida associations. Regarding folate levels, it is possible that perinatal folic acid supplementation was present and discontinued postnatally, which may have limited our ability to detect associations. For the spina bifida findings, we also do not have the ability to determine whether the maternal leukocyte DNAme patterns existed prior to the spina bifida affected pregnancy, arose as an effect of the exposure, or resulted from altered postnatal lifestyle habits.

## Conclusions

Our findings indicate that individuals with a previous spina bifida birth exhibit differential leukocyte DNAme at several loci, including developmentally important genes and loci sensitive to chronic arsenic exposure. We also identified several folate-associated loci, which did not interact with offspring spina bifida status. Future research should aim to clarify whether maternal DNAme is altered as a consequence of offspring spina bifida status, or whether these alterations reflect intrinsic maternal risk factors for offspring spina bifida.

## Supplementary Information

Supplementary Material 1

Supplementary Material 2

Supplementary Material 3

## Data Availability

Data will be made available to qualified investigators upon reasonable request and following appropriate institutional review board approval.

## References

[1] Avagliano L, Massa V, George TM, Qureshy S, Bulfamante G, Finnell RH. Overview on neural tube defects: from development to physical characteristics. Birth Defects Res. 2019;111(19):1455–67. 30421543 10.1002/bdr2.1380 PMC6511489

[2] Harris MJ, Juriloff DM. Mouse mutants with neural tube closure defects and their role in Understanding human neural tube defects. Birt Defects Res Clin Mol Teratol. 2007;79(3):187–210. 10.1002/bdra.20333 17177317

[3] Copp AJ, Adzick NS, Chitty LS, Fletcher JM, Holmbeck GN, Shaw GM. Spina bifida. Nat Rev Dis Primer. 2015;1:15007. 10.1038/nrdp.2015.7 PMC4898641 27189655

[4] Prevention of neural tube defects: Results of the Medical Research Council Vitamin Study. Lancet. 1991;20(8760):131–7. 1677062

[5] Honein MA, Paulozzi LJ, Mathews TJ, Erickson JD, Wong LYC. Impact of folic acid fortification of the US food supply on the occurrence of neural tube defects. JAMA. 2001;20(23):2981–6. 10.1001/jama.285.23.2981 11410096

[6] Christianson A, Howson C, Modell B. March of Dimes global report on birth defects: The hidden toll of dying and disabled children [Internet]. White Plains, NY: March of Dimes; 2006 [cited 2025 May 20]. Available from: https://www.alignmnh.org/resource/march-of-dimes-global-report-on-birth-defects-the-hidden-toll-of-dying-and-disabled-children/

[7] Kancherla V. Neural tube defects: a review of global prevalence, causes, and primary prevention. Childs Nerv Syst. 2023;39(7):1703–10. 10.1007/s00381-023-05910-7 36882610

[8] Lo A, Polšek D, Sidhu S. Estimating the burden of neural tube defects in low– and middle–income countries. J Glob Health. 2014;4(1):010402. 24976961 10.7189/jogh.04.010402 PMC4073251

[9] Kalliora C, Mamoulakis C, Vasilopoulos E, Stamatiades GA, Kalafati L, Barouni R, et al. Association of pesticide exposure with human congenital abnormalities. Toxicol Appl Pharmacol. 2018;346:58–75. 29596925 10.1016/j.taap.2018.03.025 PMC6029725

[10] Stillerman KP, Mattison DR, Giudice LC, Woodruff TJ. Environmental Exposures and Adverse Pregnancy Outcomes: A Review of the Science. Reprod Sci. 2008;15(7):631–50. 10.1177/1933719108322436 18836129

[11] Kalra S, Dewan P, Batra P, Sharma T, Tyagi V, Banerjee BD. Organochlorine pesticide exposure in mothers and neural tube defects in offsprings. Reprod Toxicol. 2016;66:56–60. 27647593 10.1016/j.reprotox.2016.09.005

[12] Langlois PH, Hoyt AT, Lupo PJ, Lawson CC, Waters MA, Desrosiers TA, et al. Maternal occupational exposure to polycyclic aromatic hydrocarbons and risk of neural tube defect-affected pregnancies. Birth Defects Res Part A Clin Mol Teratol. 2012;94(9):693–700. 10.1002/bdra.23045 PMC5048886 22807044

[13] Demir N, Başaranoğlu,Murat H, Zübeyir, Değer, İbrahim, Karaman, Kamuran, Şekeroğlu M, Ramazan et al. The relationship between mother and infant plasma trace element and heavy metal levels and the risk of neural tube defect in infants. J Matern Fetal Neonatal Med. 2019;32(9):1433–40. 10.1080/14767058.2017.1408064 29199526

[14] Eaves LA, Choi G, Hall E, Sillé FCM, Fry RC, Buckley JP, et al. Prenatal exposure to toxic metals and neural tube defects: A systematic review of the epidemiologic evidence. Environ Health Perspect. 2023;131(8):086002. 37647124 10.1289/EHP11872 PMC10467818

[15] Liu M, Yu J, Su Z, Sun Y, Liu Y, Xie Q, et al. Associations between prenatal exposure to cadmium and lead with neural tube defect risks are modified by single-nucleotide polymorphisms of fetal MTHFR and SOD2: a case–control study. Environ Health. 2021;5(1):66. 10.1186/s12940-021-00752-9 PMC8180011 34090432

[16] Jin L, Zhang L, Li Z, Liu J, meng, Ye R, Ren A. Placental concentrations of mercury, lead, cadmium, and arsenic and the risk of neural tube defects in a Chinese population. Reprod Toxicol. 2013;35:25–31. 23164984 10.1016/j.reprotox.2012.10.015

[17] Cengiz B, Söylemez F, Öztürk E, Çavdar AO. Serum zinc, selenium, copper, and lead levels in women with second-trimester induced abortion resulting from neural tube defects. Biol Trace Elem Res. 2004;97(3):225–35. 14997023 10.1385/BTER:97:3:225

[18] Wlodarczyk BJ, Bennett GD, Calvin JA, Finnell RH. Arsenic-induced neural tube defects in mice: alterations in cell cycle gene expression. Reprod Toxicol. 1996;10(6):447–54. 8946558 10.1016/s0890-6238(96)00131-1

[19] Hill DS, Wlodarczyk BJ, Finnell RH. Reproductive consequences of oral arsenate exposure during pregnancy in a mouse model. Birth Defects Res B Dev Reprod Toxicol. 2008;83(1):40–7. 18186108 10.1002/bdrb.20142

[20] Li X, Li G, Cui S, Hou Y, Li Z, Yan Z, et al. Arsenic disturbs neural tube closure involving AMPK/PKB-mTORC1-mediated autophagy in mice. Food Chem Toxicol. 2024;186:114538. 38387523 10.1016/j.fct.2024.114538

[21] Brender JD, Suarez L, Felkner M, Gilani Z, Stinchcomb D, Moody K, et al. Maternal exposure to arsenic, cadmium, lead, and mercury and neural tube defects in offspring. Environ Res. 2006;101(1):132–9. 16171797 10.1016/j.envres.2005.08.003

[22] Kwok RK, Kaufmann RB, Jakariya M. Arsenic in drinking-water and reproductive health outcomes: a study of participants in the Bangladesh Integrated Nutrition Programme. J Health Popul Nutr. 2006;24(2):190–205. 17195560

[23] Wu J, Chen G, Liao Y, Song X, Pei L, Wang J, et al. Arsenic levels in the soil and risk of birth defects: a population-based case-control study using GIS technology. J Environ Health. 2011;74(4):20–5. 22187854

[24] Ahmad SA, Khan MH. 2 - Groundwater arsenic contamination and its health effects in Bangladesh. In: Flora SJS, editor. Handbook of Arsenic Toxicology (Second Edition) [Internet]. Oxford: Academic Press; 2023 [cited 2025 Sept 3]. pp. 51–77. Available from: https://www.sciencedirect.com/science/article/pii/B9780323898478000079

[25] Mandai BK, Biswas BK, Dhar RK, Chowdhury TR, Samanta G, Basu GK et al. Groundwater Arsenic Contamination and Sufferings of People in West Bengal, India and Bangladesh. In: Sarkar B, editor. Metals and Genetics [Internet]. Boston, MA: Springer US; 1999 [cited 2025 Sept 3]. pp. 41–65. Available from: 10.1007/978-1-4615-4723-5_4

[26] UNICEF (United Nations Children’s Fund). WHO (World Health Organization) Water, Sanitation & Hygiene Section. Arsenic primer: Guidance on the investigation & mitigation of arsenic contamination [Internet]. 2018 [cited 2025 Sept 3]. Available from: https://www.who.int/publications/m/item/arsenic-primer

[27] Chakraborti D, Rahman MM, Das B, Murrill M, Dey S, Chandra Mukherjee S, et al. Status of groundwater arsenic contamination in bangladesh: A 14-year study report. Water Res. 2010;44(19):5789–802. 20684969 10.1016/j.watres.2010.06.051

[28] Jahan I, Hossain MA, Uddin SN, Choudhuri Moni S, Hassan MK, Dey SK et al. Incidence of neural tube defects in tertiary care university hospital in Bangladesh. Clin Exp Pediatr [Internet]. 2025 Apr 1 [cited 2025 May 20]; Available from: https://www.e-cep.org/journal/view.php?doi=10.3345/cep.2024.01578. 10.3345/cep.2024.01578 PMC12235336 40211864

[29] Wei C, Choma EF, Wang X, Wade CG, Hsiao YL, Bao D, et al. Comparing folic acid interventions and arsenic reduction strategies for neural tube defect prevention in Bangladesh: a systematic review and decision analysis. Birth Defects Res. 2025;117(6):e2494. 40552686 10.1002/bdr2.2494 PMC12186471

[30] Akhtar N, Karim S. Varieties of foetal congenital abnormality. Mymensingh Med J MMJ. 2012;21(4):662–7. 23134914

[31] Shuma M, Halder S, Datta B. Epidemiology of congenital anomalies among the children born in different hospitals under Sylhet division in Bangladesh- a retrospective study | Dhaka University Journal of Pharmaceutical Sciences. Dhaka Univ J Pharm Sci. 2016;14(2):225–30.

[32] Zaganjor I, Sekkarie A, Tsang BL, Williams J, Razzaghi H, Mulinare J, et al. Describing the prevalence of neural tube defects worldwide: a systematic literature review. PLoS One. 2016;11(4):e0151586. 27064786 10.1371/journal.pone.0151586 PMC4827875

[33] Mazumdar M, Ibne Hasan MOS, Hamid R, Valeri L, Paul L, Selhub J, et al. Arsenic is associated with reduced effect of folic acid in myelomeningocele prevention: a case control study in Bangladesh. Environ Health. 2015;14:34. 25885259 10.1186/s12940-015-0020-0 PMC4404044

[34] Wei CF, Mukherjee SK, Ekramullah SM, Arman DM, Islam MJ, Azim M, et al. Arsenic modifies the effect of folic acid in spina bifida prevention, a large hospital-based case-control study in Bangladesh. Environ Health. 2024;3(1):51. 10.1186/s12940-024-01091-1 PMC11145859 38831396

[35] Crider KS, Yang TP, Berry RJ, Bailey LB, Folate, Methylation DNA. A review of molecular mechanisms and the evidence for folate’s Role2. Adv Nutr. 2012;3(1):21–38. 22332098 10.3945/an.111.000992 PMC3262611

[36] Roy NK, Murphy A, Costa M. Arsenic methyltransferase and methylation of inorganic arsenic. Biomolecules 2020;10(9):1351. 10.3390/biom10091351 PMC7563989 32971865

[37] Thomas DJ. Arsenic methylation – Lessons from three decades of research. Toxicol 2021;457:152800. 10.1016/j.tox.2021.152800 PMC10048126 33901604

[38] Bozack AK, Saxena R, Gamble MV. Nutritional influences on one-carbon metabolism: effects on arsenic methylation and toxicity. Annu Rev Nutr. 2018;38:401–29. 29799766 10.1146/annurev-nutr-082117-051757 PMC6441546

[39] van der Put NMJ, Trijbels FJM, van den Heuvel LP, Blom HJ, Steegers-Theunissen RPM, Eskes TKAB, et al. Mutated methylenetetrahydrofolate reductase as a risk factor for spina bifida. Lancet. 1995;346(8982):1070–1. 7564788 10.1016/s0140-6736(95)91743-8

[40] Stover PJ, MacFarlane AJ, Field MS. Bringing clarity to the role of MTHFR variants in neural tube defect prevention123. Am J Clin Nutr. 2015;101(6):1111–2. 25971720 10.3945/ajcn.115.111088 PMC4441815

[41] Chen X, Guo J, Lei Y, Zou J, Lu X, Bao Y, et al. Global DNA hypomethylation is associated with NTD-affected pregnancy: a case-control study. Birth Defects Research Part A: Clinical and Molecular Teratology. 2010;88(7):575–81. 20641100 10.1002/bdra.20670

[42] Wang L, Wang F, Guan J, Le J, Wu L, Zou J, et al. Relation between hypomethylation of long interspersed nucleotide elements and risk of neural tube defects1234. Am J Clin Nutr. 2010;91(5):1359–67. 20164316 10.3945/ajcn.2009.28858

[43] Chang H, Zhang T, Zhang Z, Bao R, Fu C, Wang Z, et al. Tissue-specific distribution of aberrant DNA methylation associated with maternal low-folate status in human neural tube defects. J Nutr Biochem. 2011;22(12):1172–7. 21333513 10.1016/j.jnutbio.2010.10.003

[44] Stolk L, Bouwland-Both MI, van Mill NH, Verbiest MMPJ, Eilers PHC, Zhu H, et al. Epigenetic profiles in children with a neural tube defect; a case-control study in two populations. PLoS One. 2013;8(11):e78462. 24223810 10.1371/journal.pone.0078462 PMC3818348

[45] Rochtus A, Izzi B, Vangeel E, Louwette S, Wittevrongel C, Lambrechts D, et al. DNA methylation analysis of homeobox genes implicates HOXB7 hypomethylation as risk factor for neural tube defects. Epigenetics. 2015;10(1):92–101. 25565354 10.1080/15592294.2014.998531 PMC4622610

[46] Mazumdar M, Valeri L, Rodrigues EG, Hasan MOSI, Hamid R, Paul L, et al. Polymorphisms in maternal folate pathway genes interact with arsenic in drinking water to influence risk of myelomeningocele. Birth Defects Res A Clin Mol Teratol. 2015;103(9):754–62. 26250961 10.1002/bdra.23399 PMC4565773

[47] Tindula G, Mukherjee SK, Ekramullah SM, Arman D, Biswas S, Islam J, et al. Parental metal exposures as potential risk factors for spina bifida in Bangladesh. Environ Int. 2021;157:106800. 34358915 10.1016/j.envint.2021.106800 PMC9008873

[48] Aryee MJ, Jaffe AE, Corrada-Bravo H, Ladd-Acosta C, Feinberg AP, Hansen KD, et al. Minfi: a flexible and comprehensive bioconductor package for the analysis of infinium DNA methylation microarrays. Bioinformatics. 2014;30(10):1363–9. 24478339 10.1093/bioinformatics/btu049 PMC4016708

[49] Murat K, Grüning B, Poterlowicz PW, Westgate G, Tobin DJ, Poterlowicz K. Ewastools: infinium human methylation BeadChip pipeline for population epigenetics integrated into galaxy. Gigascience. 2020;9(5):giaa049. 32401319 10.1093/gigascience/giaa049 PMC7219210

[50] Leek JT, Johnson WE, Parker HS, Jaffe AE, Storey JD. The Sva package for removing batch effects and other unwanted variation in high-throughput experiments. Bioinformatics. 2012;28(6):882–3. 22257669 10.1093/bioinformatics/bts034 PMC3307112

[51] Kaur D, Lee SM, Goldberg D, Spix NJ, Hinoue T, Li HT et al. Comprehensive evaluation of the infinium human methylationepic v2 BeadChip. Epigenetics Commun 2023;3(1):6. 10.1186/s43682-023-00021-5 PMC10919401 38455390

[52] Peters TJ, Meyer B, Ryan L, Achinger-Kawecka J, Song J, Campbell EM, et al. Characterisation and reproducibility of the humanmethylationepic v2.0 BeadChip for DNA methylation profiling. BMC Genomics. 2024;25(1):251. 38448820 10.1186/s12864-024-10027-5 PMC10916044

[53] Salas L, Koestler D. FlowSorted.Blood.EPIC: Illumina EPIC data on immunomagnetic sorted peripheral adult blood cells [Internet]. 2024. Available from: https://github.com/immunomethylomics/FlowSorted.Blood.EPIC

[54] Salas LA, Zhang Z, Koestler DC, Butler RA, Hansen HM, Molinaro AM, et al. Enhanced cell Deconvolution of peripheral blood using DNA methylation for high-resolution immune profiling. Nat Commun. 2022;13(1):761. 35140201 10.1038/s41467-021-27864-7 PMC8828780

[55] Ritchie ME, Phipson B, Wu D, Hu Y, Law CW, Shi W, et al. Limma powers differential expression analyses for RNA-sequencing and microarray studies. Nucleic Acids Res. 2015;43(7):e47–47. 25605792 10.1093/nar/gkv007 PMC4402510

[56] Xie C, Leung YK, Chen A, Long DX, Hoyo C, Ho SM. Differential methylation values in differential methylation analysis. Bioinformatics. 2019;35(7):1094–7. 30184051 10.1093/bioinformatics/bty778 PMC6449748

[57] Singh G, Hamdan H, Singh V. Clinical utility of serum folate measurement in tertiary care patients: argument for revising reference range for serum folate from 3.0 ng/mL to 13.0 ng/mL. Pract Lab Med. 2015;1:35–41. 28932797 10.1016/j.plabm.2015.03.005 PMC5597708

[58] Peters TJ, Buckley MJ, Statham AL, Pidsley R, Samaras K, Lord RV et al. IDe novo identification of differentially methylated regions in the human genome. Epigenetics Chromatin. 2015;8:6. 10.1186/1756-8935-8-6. 10.1186/1756-8935-8-6 PMC4429355 25972926

[59] Phipson B, Maksimovic J, Oshlack A. MissMethyl: an R package for analyzing data from illumina’s HumanMethylation450 platform. Bioinforma Oxf Engl. 2016;32(2):286–8. 10.1093/bioinformatics/btv560 26424855

[60] Rochtus A, Winand R, Laenen G, Vangeel E, Izzi B, Wittevrongel C, et al. Methylome analysis for spina bifida shows SOX18 hypomethylation as a risk factor with evidence for a complex (epi)genetic interplay to affect neural tube development. Clin Epigenetics. 2016;8:108. 27757173 10.1186/s13148-016-0272-8 PMC5064967

[61] Bozack AK, Boileau P, Wei L, Hubbard AE, Sillé FCM, Ferreccio C, et al. Exposure to arsenic at different life-stages and DNA methylation meta-analysis in buccal cells and leukocytes. Environ Health. 2021;9:20:79. 10.1186/s12940-021-00754-7 PMC8272372 34243768

[62] Argos M, Chen L, Jasmine F, Tong L, Pierce BL, Roy S, et al. Gene-specific differential DNA methylation and chronic arsenic exposure in an epigenome-wide association study of adults in Bangladesh. Environ Health Perspect. 2015;123(1):64–71. 25325195 10.1289/ehp.1307884 PMC4286273

[63] Wang C, Xu Z, Qiu X, Wei Y, Peralta AA, Yazdi MD, et al. Epigenome-wide DNA methylation in leukocytes and toenail metals: the normative aging study. Environ Res. 2022;217:114797. 36379232 10.1016/j.envres.2022.114797 PMC9825663

[64] Demanelis K, Argos M, Tong L, Shinkle J, Sabarinathan M, Rakibuz-Zaman M, et al. Association of arsenic exposure with whole blood DNA methylation: an Epigenome-Wide study of Bangladeshi adults. Environ Health Perspect. 2019;127(5):057011. 31135185 10.1289/EHP3849 PMC6791539

[65] Fragoso-Bargas N, Page,Christian M, Joubert, Bonnie R, London SJ et al. Lee-Ødegård, Sindre, Opsahl, Julia O,. Epigenome-Wide Association Study of Serum Folate in Maternal Peripheral Blood Leukocytes. Epigenomics. 2023;15(1):39–52. 10.2217/epi-2022-0427 PMC10072132 36974632

[66] Mandaviya PR, Joehanes R, Brody J, Castillo-Fernandez JE, Dekkers KF, Do AN, et al. Association of dietary folate and vitamin B-12 intake with genome-wide DNA methylation in blood: a large-scale epigenome-wide association analysis in 5841 individuals. Am J Clin Nutr. 2019;110(2):437–50. 31165884 10.1093/ajcn/nqz031 PMC6669135

[67] Joubert BR, den Dekker HT, Felix JF, Bohlin J, Ligthart S, Beckett E, et al. Maternal plasma folate impacts differential DNA methylation in an epigenome-wide meta-analysis of newborns. Nat Commun. 2016;7:10577. 26861414 10.1038/ncomms10577 PMC4749955

[68] Bozack AK, Domingo-Relloso A, Haack K, Gamble MV, Tellez-Plaza M, Umans JG, et al. Locus-Specific differential DNA methylation and urinary arsenic: an Epigenome-Wide association study in blood among adults with Low-to-Moderate arsenic exposure. Environ Health Perspect. 2020;30(6):067015. 10.1289/EHP6263 PMC7534587 32603190

[69] Price EM, Peñaherrera MS, Portales-Casamar E, Pavlidis P, Van Allen MI, McFadden DE et al. Profiling placental and fetal DNA methylation in human neural tube defects. Epigenetics Chromatin [Internet]. 2016 Dec [cited 2018 Apr 9];9(1). Available from: http://www.epigeneticsandchromatin.com/content/9/1/6 10.1186/s13072-016-0054-8 PMC4756451 26889207

[70] Huang Y, Lin S, Jin L, Wang L, Ren A. Decreased global DNA hydroxymethylation in neural tube defects: association with polycyclic aromatic hydrocarbons. Epigenetics. 2019;14(10):1019–29. 31179819 10.1080/15592294.2019.1629233 PMC6691991

[71] Hubert KA, Wellik DM. Hox genes in development and beyond. Dev Camb Engl. 2023;150(1):dev192476. 10.1242/dev.192476 PMC10216783 36645372

[72] Yu J, Wang L, Pei P, Li X, Wu J, Qiu Z, et al. Reduced H3K27me3 leads to abnormal hox gene expression in neural tube defects. Epigenetics Chromatin. 2019;12:76. 31856916 10.1186/s13072-019-0318-1 PMC6921514

[73] Figueiredo AL, Maczkowiak F, Borday C, Pla P, Sittewelle M, Pegoraro C, et al. PFKFB4 control of AKT signaling is essential for premigratory and migratory neural crest formation. Dev Camb Engl. 2017;144(22):4183–94. 10.1242/dev.157644 29038306

[74] Zhang J, Yang L, Sun Y, Zhang L, Wang Y, Liu M, et al. Up-regulation of miR-10a-5p expression inhibits the proliferation and differentiation of neural stem cells by targeting Chl1. Acta Biochim Biophys Sin. 2024;5(10):1483–97. 10.3724/abbs.2024078 PMC11532229 38841745

[75] Mazumdar M. Does arsenic increase the risk of neural tube defects among a highly exposed population? A new case-control study in Bangladesh. Birth Defects Res. 2017;109(2):92–8. 27801974 10.1002/bdra.23577 PMC5388562

[76] Denny KJ, Coulthard LG, Jeanes A, Lisgo S, Simmons DG, Callaway LK, et al. C5a receptor signaling prevents folate Deficiency-Induced neural tube defects in mice. J Immunol Baltim Md 1950. 2013;190(7):3493–9. 10.4049/jimmunol.1203072 PMC3608813 23420882

[77] Perrier F, Viallon V, Ambatipudi S, Ghantous A, Cuenin C, Hernandez-Vargas H, et al. Association of leukocyte DNA methylation changes with dietary folate and alcohol intake in the EPIC study. Clin Epigenetics. 2019;11(1):57. 30940212 10.1186/s13148-019-0637-x PMC6444439

[78] Gonseth S, Roy R, Houseman EA, de Smith AJ, Zhou M, Lee ST, et al. Periconceptional folate consumption is associated with neonatal DNA methylation modifications in neural crest regulatory and cancer development genes. Epigenetics. 2015;10(12):1166–76. 26646725 10.1080/15592294.2015.1117889 PMC4844202

[79] Ameer SS, Engström K, Hossain MB, Concha G, Vahter M, Broberg K. Arsenic exposure from drinking water is associated with decreased gene expression and increased DNA methylation in peripheral blood. Toxicol Appl Pharmacol. 2017;321:57–66. 28242323 10.1016/j.taap.2017.02.019

[80] Signes-Pastor AJ, Gutiérrez-González E, García-Villarino M, Rodríguez-Cabrera FD, López-Moreno JJ, Varea-Jiménez E, et al. Toenails as a biomarker of exposure to arsenic: A review. Environ Res. 2021;195:110286. 33075355 10.1016/j.envres.2020.110286 PMC7987585

[81] Court F, Tayama C, Romanelli V, Martin-Trujillo A, Iglesias-Platas I, Okamura K, et al. Genome-wide parent-of-origin DNA methylation analysis reveals the intricacies of human imprinting and suggests a germline methylation-independent mechanism of establishment. Genome Res. 2014;24(4):554–69. 24402520 10.1101/gr.164913.113 PMC3975056

[82] Hoyo C, Murtha AP, Schildkraut JM, Jirtle R, Demark-Wahnefried W, Forman MR, et al. Methylation variation at IGF2 differentially methylated regions and maternal folic acid use before and during pregnancy. Epigenetics. 2011;6(7):928–36. 21636975 10.4161/epi.6.7.16263 PMC3154433

[83] Amarasekera M, Martino D, Ashley S, Harb H, Kesper D, Strickland D, et al. Genome-wide DNA methylation profiling identifies a folate-sensitive region of differential methylation upstream of ZFP57-imprinting regulator in humans. FASEB J. 2014;28(9):4068–76. 24891518 10.1096/fj.13-249029 PMC5395735

