# Supplementary material for: Associations of arsenic exposure and folate in maternal leukocyte DNA methylation: a case-control study of mothers with spina-bifida affected children
Source: Environ Health. 2026 Jan 16;25:13. doi: 10.1186/s12940-025-01254-8 (PMC12896015; doi:10.1186/s12940-025-01254-8)
Supplement: Supplementary file 1 — Supplementary Material 1 [file 12940_2025_1254_MOESM1_ESM.pdf]

Supplementary Information for:

Associations of arsenic exposure and folate in maternal leukocyte DNA methylation: A case-control study of mothers with spina-bifida affected children

Amy M Inkster[a,*], Anne K Bozack[a], Bernardo Lemos[b,c], Tabitha Lumour-Mensah[d], Sudipta Kumar Mukherjee[e], Shekh Muhammad Ekramullah[e], DM Arman[e], Joynul Islam[f], Xingyan Wang[g], Liming Liang[g], Richard H Finnell[h,i], Maitreyi Mazumdar[4,d,j,k,†], Andres Cardenas[a,†]

Supplementary Tables

Supplementary Table 1. (Externally attached table – CpGs that reach FDR significance in 6 EWAS models)

Supplementary Table 2. Differentially-methylated regions (DMRs) associated with spina bifida case/control status (Model 1), and the interaction of spina bifida status and arsenic (Model 2). All DMRs were identified based on individual CpG sites satisfying an FDR < 0.05. Stouffer FDR values are calculated by combining the raw p values from individual CpGs and FDR adjusting after combination.

DMR ID	No. CpGs	Location (hg38)	Width (bp)	Stouffer FDR	Gene(s)
**Spina bifida main effect (Model 1a)**					
1	2	chr20:50,284,883	13	1.68E-26	
2	4	chr3:50,612,563	770	1.54E-20	*MAPKAP3*
3	14	chr1:24,964,894	688	2.73E-20	*HOXB3, HOXB-AS3, AC103702.1, MIR10A*
4	21	chr17:81,821,352	1097	3.38E-19	*MCRIP1*
5	4	chr17:59,838,304	109	7.45E-16	*CCR7*
6	4	chr17:40,560,550	492	1.41E-15	*RUNX3*
7	6	chr16:3,065,551	258	1.41E-15	*IL32*
8	13	chr17:48,579,181	1628	5.48E-15	
9	2	chr12:122,004,950	8	6.56E-14	*VMP1*
**Arsenic main effect (Model 1b)**					
1	4	chr2:27,307,802	501	3.18E-15	*UCN*
**Folate main effect (Model 1c)**					
1	8	chr6:36,024,723	192	2.21E-18	*SLC26A8*
2	7	chr12:4,379,583	407	6.70E-17	*AC008012.1, FGF23*
3	10	chr1:111,200,292	624	3.42E-13	*CHI3L2, DENND2D*
4	2	chr12:14,260,647	110	1.08E-12	
5	5	chr17:80,774,134	300	2.28E-12	*RPTOR*
**Spina bifida*Arsenic interaction effect (Model 2)**					
1	2	chr19:11,374,579	13	3.03E-24	
2	4	chr19:11,374,579	770	4.42E-19	*MAPKAP3*
3	4	chr19:11,374,579	492	1.14E-17	*CCR7*
4	4	chr19:11,374,579	109	1.84E-17	*VMP1*
5	15	chr19:11,374,579	1032	1.86E-17	*RUNX3*
6	19	chr19:11,374,579	798	1.94E-16	*MCRIP1*
7	2	chr19:11,374,579	8	1.09E-13	
8	6	chr19:11,374,579	641	3.79E-09	*HOXB3, HOXB-AS3, AC103702.1, MIR10A*
**Spina bifida*Folate interaction effect (Model 3)**					
No FDR significant associations.					
**Arsenic*Folate interaction effect (Model 4)**					
1	7	chr8:103,371,185	559	4.46E-10	*CTHRC1*
2	11	chr6:116,279,475	1026	1.92E-09	*DSE, TSPYL1*
3	8	chr17:19,748,318	353	8.52E-09	*ALDH3A1*
4	4	chr18:12,777,646	385	9.95E-09	
5	4	chr4:1,212,126	237	1.74E-08	*CTBP1-AS, CTBP1*
6	2	chr9:37,853,488	201	2.26E-08	*DCAF10, AL138752.2*
7	5	chr17:82,583,861	382	3.54E-08	*FOXK2*
8	2	chr12:101,713,326	7	6.52E-08	*CHPT1*

9	6	chr12:1,820,165	444	1.00E-07	*LRTM2, CACNA2D4*
10	3	chr8:1,416,883	454	1.08E-07	*DLGAP2*
11	3	chr16:57,884,042	150	3.11E-07	*CNGB1*
12	3	chr7:14,747,772	7	4.40E-07	*DGKB*
13	2	chr10:3,924,390	46	3.17E-06	*LINC02660*
14	2	chr11:77,337,237	158	3.86E-06	*PAK1*
15	2	chr19:54,420,458	157	4.41E-06	*TTYH1*
16	2	chr5:142,982,264	295	6.47E-06	*ARHGAP26*
17	2	chr6:36,391,553	38	9.29E-06	*ETV7-AS1, PXT1*
18	2	chr12:7,132,884	184	1.40E-04	*CLSTN3*
19	3	chr17:81,131,960	140	2.24E-04	*AATK*
20	2	chr1:168,499,485	415	2.42E-04	
21	2	chr11:27,473,619	228	7.04E-04	*LGR4-AS1*
22	3	chr2:27,307,802	215	1.08E-03	*UCN*
23	8	chr2:37,671,880	754	1.80E-02	*CDC42EP3*
24	4	chr2:151,734,789	665	1.20E-01	
25	3	chr16:1,262,980	100	2.51E-01	*PRSS29P*

Supplementary Figures

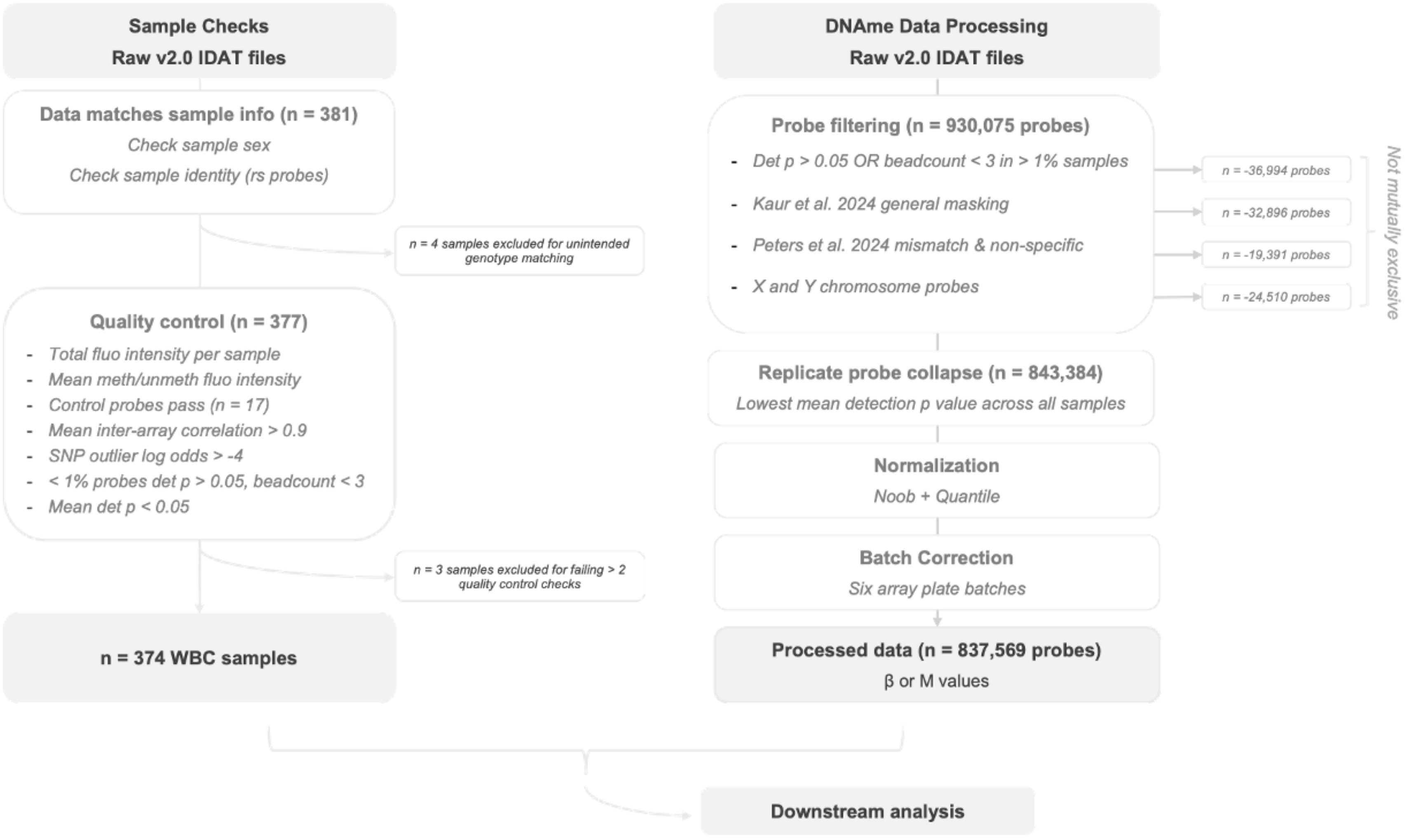

Supplementary Figure 1. Workflow diagram for sample quality control and DNAme data processing. Fluo refers to fluorescence, meth/unmeth refer to methylated and unmethylated fluorescence intensities, det p refers to detection p value.

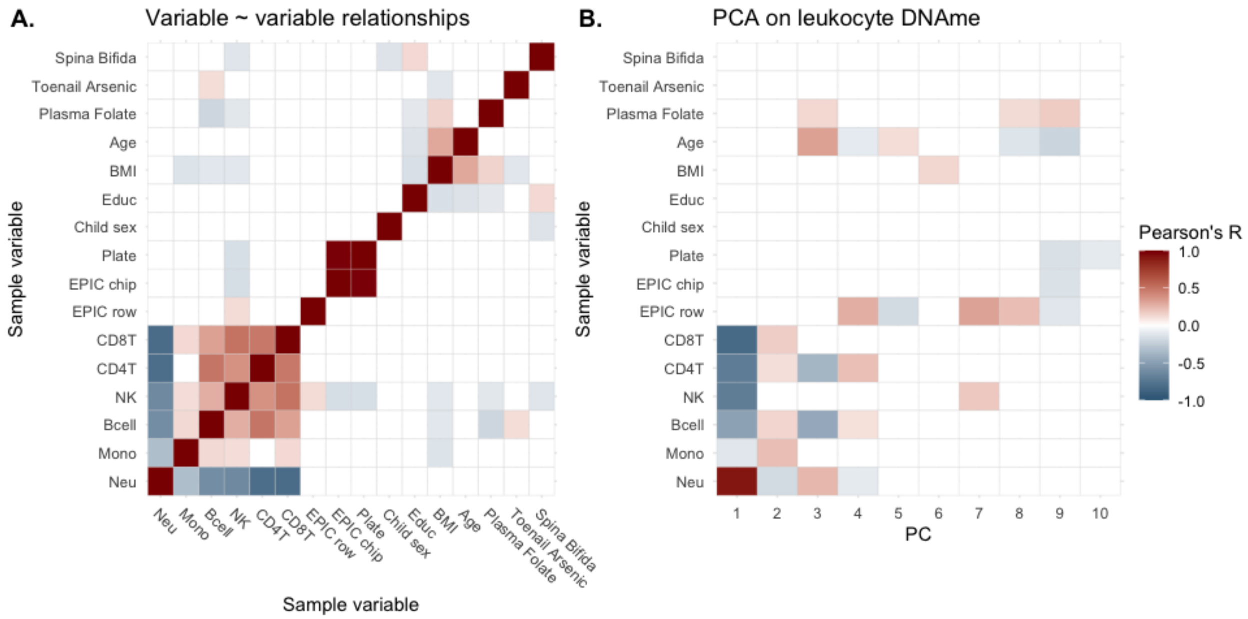

Supplementary Figure 2. Assessment of dataset characteristics. For both plots cells are shaded by R^2 values where relationships are significant ($p < 0.05$), positive relationships are shown in red, negative in blue. **(A)** Variable-variable relationships among the 374 inclusion samples. **(B)** Principal components analysis heatmap showing relationships between the first 10 PCs of the DNAme data (after processing and batch correction) and sample variables.

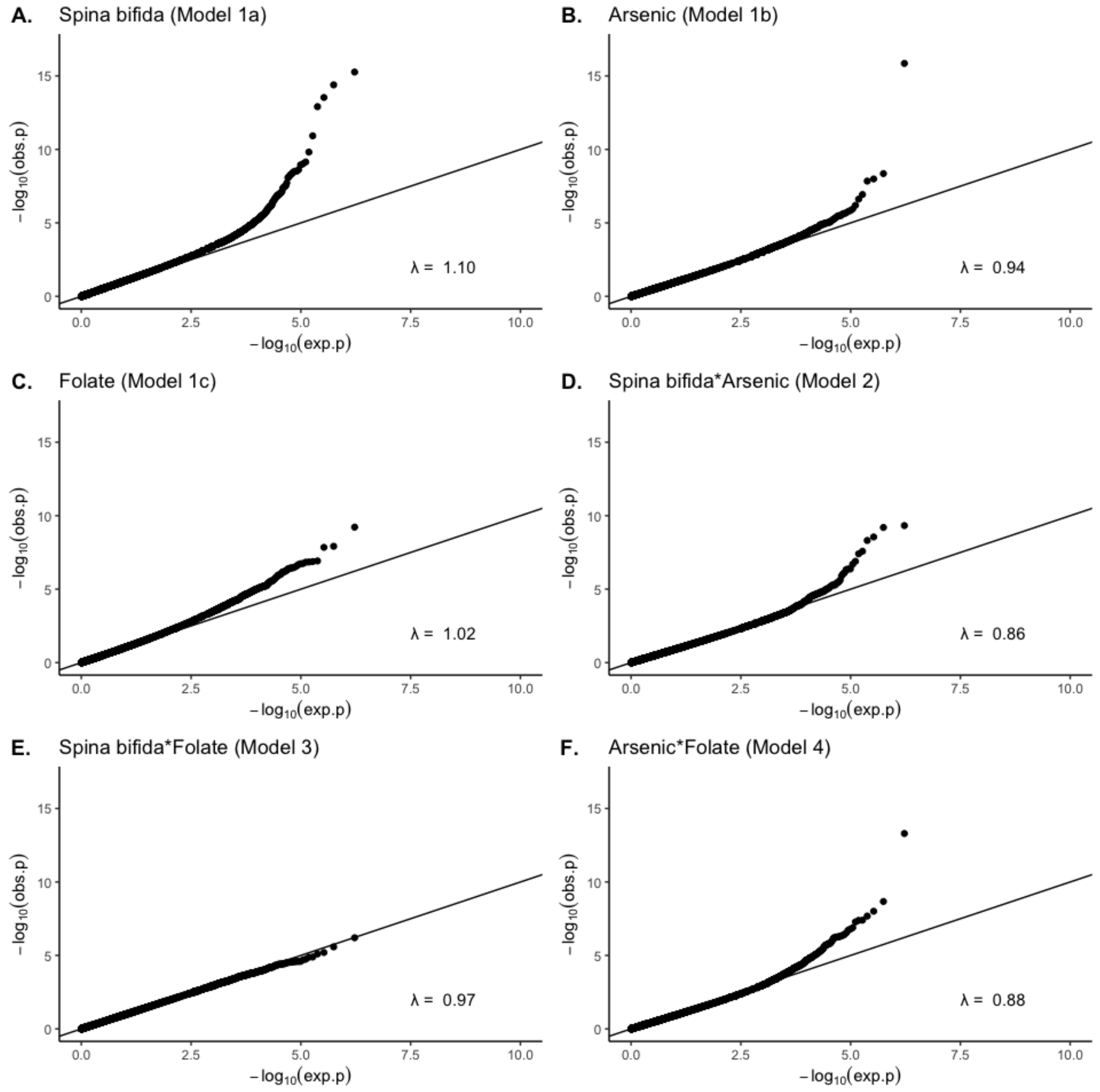

Supplementary Figure 3. Evaluation of genomic inflation across epigenome-wide linear models. For all plots, quantile-quantile plots depict the -log10 of the observed nominal p values (obs.p, Y axis) plotted against a uniform distribution of expected p values (exp.p, X axis). The lambda (λ) value is shown in the bottom right of the plot, calculated as a metric of inflation using the observed p values. The standard error of the lambda value is also provided, and was calculated using a bootstrapping method, resampling the observed p values with replacement 100 times. The bootstrapped observed median is divided by the expected median of the chi-squared distribution under the null, and standard error reported corresponds to the standard deviation of the bootstrapped lambda values. Any genes overlapping CpGs that reach genome-wide significance are labelled, overlapping transcripts are separated by semi colons. **(A) S**pina bifida status, Model 1. **(B)** Interaction of spina bifida status and toenail arsenic (μg/g), Model 2. **(C)** Interaction of spina bifida status and plasma folate (ng/mL), Model 3. **(D)** Main effect of toenail arsenic (μg/g), Model 4. **(E)** Main effect of plasma folate (ng/mL), Model 5. **(F)** Interaction of toenail arsenic (μg/g) and plasma folate (ng/mL), Model 6.

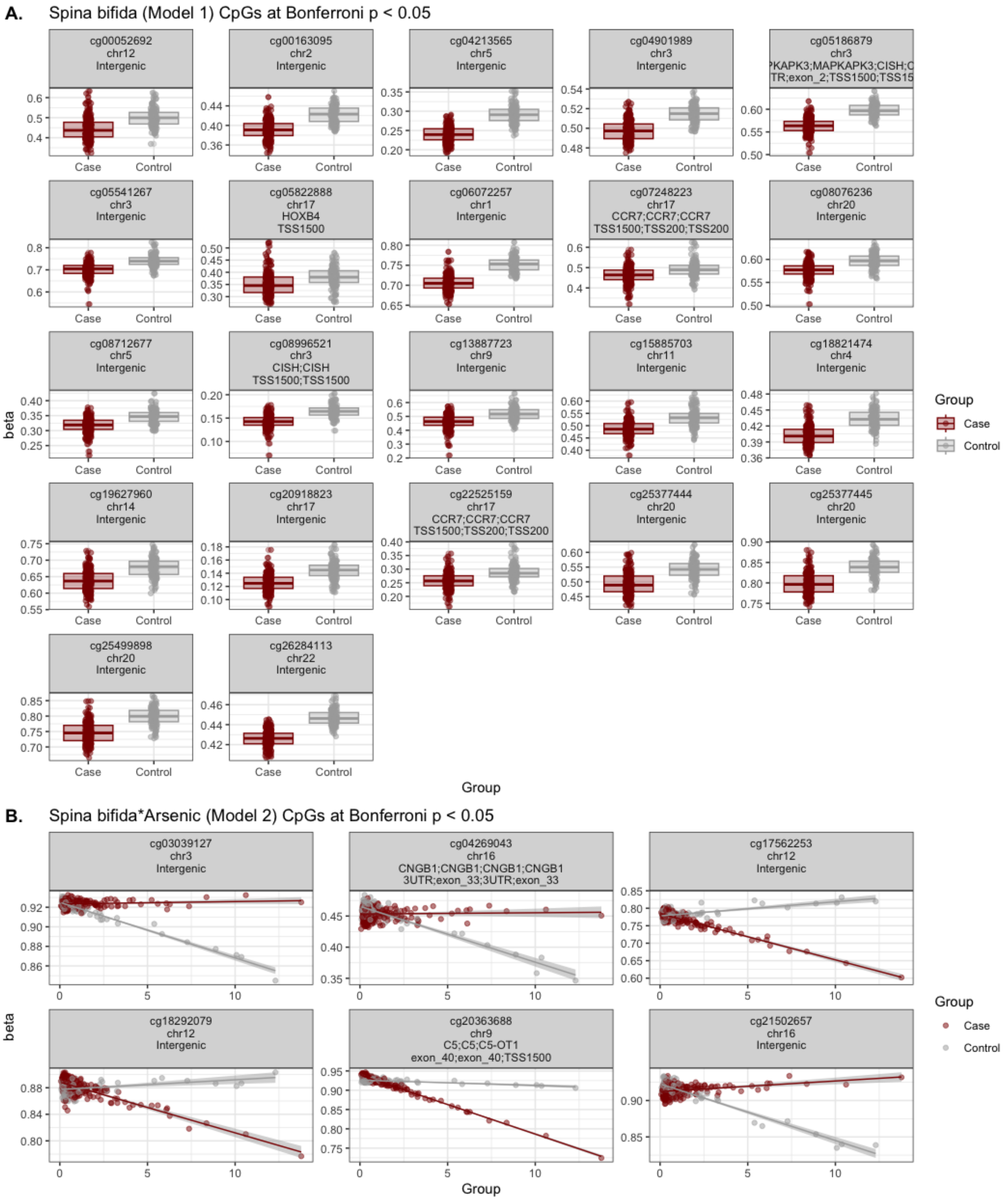

Supplementary Figure 4. Spina-bifida-associated DNAme at the top hits from Models 1 and 2. (A) Model 1 Bonferroni p top hits. (B) Model 2 Bonferroni p top hits.

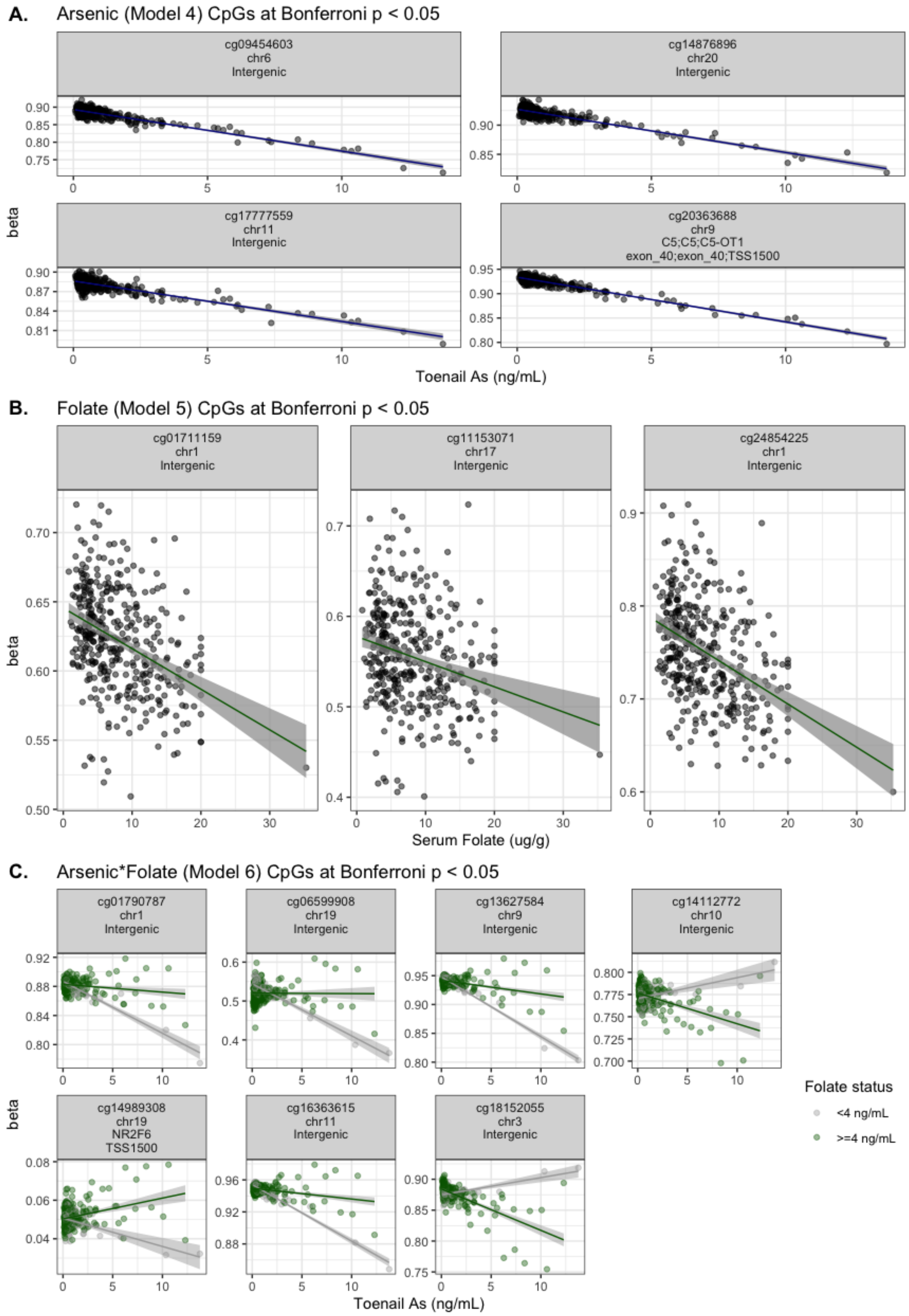

Supplementary Figure 5. Arsenic and folate-associated DNAme at the top CpG hits from Models 4, 5, and 6. (A) Model 4 Bonferroni p top hits. (B) Model 5 Bonferroni p top hits. (C) Model 6 Bonferroni p top hits.
